# Dissecting the bidirectional associations between the progression of gastrointestinal and endocrine diseases

**DOI:** 10.3389/fendo.2025.1538603

**Published:** 2025-05-06

**Authors:** Hongyan Liu, Shucheng Si, Hua Zhang, Siyan Zhan

**Affiliations:** ^1^ Research Center of Clinical Epidemiology, Peking University Third Hospital, Beijing, China; ^2^ Peking University Health Science Center, Beijing, China; ^3^ Key Laboratory of Epidemiology of Major Diseases (Peking University), Ministry of Education, Beijing, China; ^4^ Department of Epidemiology and Biostatistics, School of Public Health, Peking University, Beijing, China; ^5^ Institute for Artificial Intelligence, Peking University, Beijing, China

**Keywords:** gastrointestinal diseases, endocrine diseases, comorbidities, bidirectional relationship, UK Biobank (UKB)

## Abstract

**Backgrounds:**

The widespread intrinsic link between gastrointestinal and endocrine diseases was poorly understood. We aimed to dissect the bidirectional association in the progression of gastrointestinal with endocrine diseases, either the overall, individual, or comorbidities with each other.

**Methods:**

A bidirectional-designed prospective cohort included 481841 and 452858 participants free of gastrointestinal and endocrine diseases at baseline in the UK Biobank. Multivariable Cox proportional hazard models were used to estimate the hazard ratios (HRs) and 95% confidence interval (CI) for incident endocrine diseases according to gastrointestinal disease status or the number of gastrointestinal comorbidities, and vice versa.

**Results:**

Overall gastrointestinal disease was associated with an increased risk of incident endocrine diseases (HR, 1.22; 95% CI, 1.19-1.25), and conversely, overall endocrine disease also increased the risk of total gastrointestinal diseases (HR, 1.48; 95% CI, 1.44-1.53). For specific diseases, extensive bidirectional associations were observed between type 2 diabetes and six gastrointestinal diseases (gastritis and duodenitis, irritable bowel syndrome, gastrointestinal hemorrhage, dyspepsia, duodenal ulcer, and gastric ulcer), thyroid disorders, and five gastrointestinal diseases (gastritis and duodenitis, irritable bowel syndrome, dyspepsia, malabsorption, and ulcerative colitis), hyperparathyroidism and three gastrointestinal diseases (gastritis and duodenitis, gastrointestinal hemorrhage, and duodenal ulcer), etc. The risk of overall endocrine (HR, 1.14; 95% CI, 1.12-1.16) and gastrointestinal diseases (HR, 1.50; 95% CI, 1.46-1.55) increased with per-comorbidity increasing. This trend was similarly observed for most individual diseases.

**Conclusions:**

We observed an extensive bidirectional association in overall, specific, and number of comorbidities between gastrointestinal and endocrine diseases.

## Introduction

Endocrine diseases and gastrointestinal diseases seriously affect the population’s health and cause a serious disease burden. From 2000 to 2019, the prevalence rates increased for all endocrine diseases (1) and the age-standardized death rates also showed an increasing trend (2), which implied high healthcare costs and more burden in the future. Besides, gastrointestinal diseases are also major contributors to the disease burden in recent decades. For incidence, gastrointestinal cancers accounted for 36.2% of neoplasms-related deaths (3) and the cases of inflammatory bowel disease (IBD) also reached 6.8 million reported in 2017 according to the Global Burden of Disease study (4, 5). Therefore, there was an urgent need to adopt prevention strategies to reduce negative health outcomes globally.

Except for food digestion and absorption, the gut hormones, produced by the gastrointestinal tract, a major endocrine organ, are also involved in the regulation of endocrine and metabolic processes (6). Previous studies have suggested that there may be an intrinsic link between endocrine diseases and gastrointestinal diseases. A previous observational study reported that type 2 diabetes (T2D) was associated with increased risks of a wide range of gastrointestinal outcomes, suggesting the importance of early detection and prevention of gastrointestinal disorders among patients with T2D (7). On the other hand, it was also appreciated that the gastrointestinal tract plays an important role in glucose homeostasis (8, 9). In addition, the gastrointestinal disorders-celiac disease was reported to be linked with several endocrine disorders, including type 1 diabetes and thyroid disorders (10). Meanwhile, the thyroid disorders revealed a potentially higher comorbidity rate with some gastrointestinal diseases, such as autoimmune thyroid disease with celiac disease (11), Graves’ disease with IBD (12), ulcerative colitis with Hashimoto’s thyroiditis (11), et al. These connections may be driven through some potential biological pathways such as the brain-gut axis and thyroid-gut axis (13, 14). However, previous studies have only explored associations between several specific diseases. A systemic investigation of the complex relationship between endocrine and gastrointestinal diseases, and their directions of progression, was still lacking.

To bridge this knowledge gap, we designed a bidirectional prospective framework to investigate the relationship between overall and specific endocrine diseases and gastrointestinal diseases through a large population-based cohort, which had a median follow-up period of more than 13 years. This comprehensive study may have important implications for early detection and identification of high-risk populations to intercept the potential inter-progression of endocrine diseases with gastrointestinal diseases.

## Methods

### Study population and study design

This study used the UK Biobank cohort, a population-based large prospective cohort including over 500,000 participants (aged 38–73 years) recruited between 2006 and 2010 from 22 assessment centers across the United Kingdom. Participants completed a self-administered touchscreen questionnaire on sociodemographic characteristics, lifestyle exposures, medical history, medication use, and physical measurements at recruitment. Extensive medical information was collected during both recruitment and the longitudinal follow-up period. Additional details about this cohort can be found elsewhere or on the official website (www.ukbiobank.ac.uk). To analyze the association of gastrointestinal diseases with incident endocrine diseases, we included 481841 participants after excluding participants who had prevalent endocrine diseases at baseline. Similarly, 452858 participants were available when analyzing the association between endocrine diseases and risk of gastrointestinal diseases, and participants who had prevalent gastrointestinal diseases at baseline were excluded. All participants included provided signed informed consent.

### Ascertainment of gastrointestinal diseases and endocrine disease

The diseases were ascertained based on diagnoses derived from the self-reported questionnaire, and linked data from primary care and hospital inpatient registers, using the International Classification of Diseases tenth edition (ICD-10). For gastrointestinal diseases, besides considering all of them as one outcome, we additionally identified 9 individual diagnoses of gastrointestinal non-neoplastic diseases (gastric ulcer, duodenal ulcer, gastritis and duodenitis, dyspepsia, Crohn’s disease, ulcerative colitis, irritable bowel syndrome, intestinal malabsorption, and gastrointestinal hemorrhage) and 3 individual diagnoses of gastrointestinal neoplastic diseases (gastric cancer, small intestine cancer, and colorectal cancer). For, endocrine diseases, besides considering all of them as one outcome, we additionally identified 12 individual diagnoses of endocrine diseases (hypothyroidism, hyperthyroidism, thyroiditis, T2D, hypoparathyroidism, hyperparathyroidism, hyperpituitarism, hypopituitarism, Cushing syndrome, hyperaldosteronism, ovarian dysfunction, and testicular dysfunction), and 9 subtypes of endocrine diseases according to the involved gland (thyroid, pancreatic, parathyroid, pituitary, adrenal, genital glands) or axis (hypothalamic-pituitary-adrenal [HPA], hypothalamic-pituitary-thyroid [HPT] and hypothalamic-pituitary-gonadal [HPG] axis). A detailed description of the disease definition is provided in **Supplementary Tables S1**, **S2**.

### Ascertainment of covariates

The covariates used in this study included baseline demographics, lifestyle behaviors, and social isolation. Baseline demographics included age, sex, body mass index (BMI), waist-to-hip ratio (WHR), the Townsend Deprivation Index (a composite index reflecting socioeconomic status), employment status (working, retiring, or other), qualifications (with or without university/college degree), and ethnicity. Lifestyle behaviors included smoking status (current, previous smoker or not), alcohol consumption (current, previous drinker or not), alcohol consumption frequency (daily or almost daily, 3-4 times a week, 1-2 times a week, 1-3 times a month, special occasions only, and none), total physical activity (total metabolic equivalent task minutes per week), and healthy diet (greater intake of fruits, vegetables, whole grains, milk, fish or shellfish, dairy products, vegetable oils, and water, along with decreased or no intake of refined grains, processed and unprocessed meats, and sugar-sweetened beverages) (15). Social isolation was assessed by three aspects including number in household, friend/family visits, and leisure/social activities (16). For each aspect, low risk counted 0 score, and high risk counted 1. High-risk aspects were defined as living alone, friends/family visits occurring fewer times than once a month, and no participation in leisure/social activities. Social isolation was defined if the total social isolation score ≥ 2. We also gathered information on vitamin supplements, overall health status, C-reactive protein (CRP) level, and medication use (**Supplementary Table S3**).

### Statistical analysis

All participants were followed up from the date of recruitment until the date of disease diagnosis, death, or the end of the study period (December 17, 2022). Baseline characteristics of the study participants were summarized as mean with SD or median (interquartile range) for continuous variables and frequency and percentage for categorical variables. Cox proportional hazard models were used to estimate the hazard ratios (HRs) and 95% confidence interval (CI) for incident endocrine diseases associated with gastrointestinal diseases or the number of gastrointestinal comorbidities. As shown in supplement **Supplementary Figure S1**, the design and analysis were divided into two parts. Firstly, we constructed a cohort for the baseline gastrointestinal diseases (excluding the baseline endocrine diseases) and performed Cox regressions for the risk of future endocrine diseases. Then, for the reverse association, we constructed a cohort for the baseline endocrine diseases (excluding the baseline gastrointestinal diseases) and performed another Cox regression for the risk of future gastrointestinal diseases. The proportional hazards assumption was tested by creating a time-dependent variable and no violation was found. In above Cox regression models, we adjusted for the baseline age (continuous), sex (male or female), ethnicity (White people, Black people, Asian, and other), BMI (continuous), Townsend deprivation index (continuous), employment status (working, retired, or other), qualifications (college attendance or not), physical activity (continuous), smoking status (current, previous, or never), alcohol consumption (current, previous, or never), adherence to a healthy diet (yes or no), social isolation (yes or no), and CRP (continuous).

A parallel analysis was conducted to evaluate the association of endocrine diseases or the number of endocrine comorbidities with the risk of gastrointestinal diseases, and vice versa.

In sensitivity analysis, to investigate whether the bidirectional association between gastrointestinal diseases and endocrine diseases differed by subgroups, stratification analyses were further performed for the main analyses according to sex (men or women), age (<60y or ≥60y), smoking status (current smokers or not), drinking status (current drinkers or not), BMI (obese or not), and medicine use (yes or no). To assess whether the identified relationships change over time, sensitivity analysis was conducted by excluding the participants who developed within 1 year of follow-up. To confirm the accuracy of our results, sensitivity analysis was further conducted by excluding participants who had missing data on covariates or participants with new-onset gastrointestinal diseases (as exposure group) or endocrine diseases (as exposure group) during follow-up. All analyses were performed using R (version 4.2.2) software. The statistical tests were two-sided, and the significance threshold was 0.05.

## Results

### Baseline characteristics

**Table 1** presents the baseline characteristics of 481841 participants stratified by gastrointestinal disease status in detail. Among those, the mean age was 56.41 (SD: 8.10) years; 54% of participants were women. Compared with participants without gastrointestinal diseases, participants with gastrointestinal diseases were more likely to be smokers, have lower socioeconomic status, lower physical activity, and higher social isolation. They were less likely to work and have a college or university education. **Supplementary Table S4** presents the baseline characteristics of 452858 participants stratified by endocrine disease status in detail.

**Table 1:** Baseline characteristics of study participants according to baseline gastrointestinal diseases status.

Baseline characteristics	Overall	Participants with gastrointestinal diseases	Participants without gastrointestinal diseases
Number of participants	481841	44455	437386
Age (years)	56.41 (8.10)	58.44 (7.68)	56.20 (8.11)
Female (%)	261392 (54.25)	25024 (56.29)	236368 (54.04)
WHR	0.87 (0.09)	0.88 (0.09)	0.87 (0.09)
BMI (kg/m2)	27.32 (4.71)	27.75 (4.94)	27.28 (4.68)
Townsend index	-1.32 (3.08)	-0.87 (3.28)	-1.37 (3.06)
Employment status (%)			
Working	282930 (58.72)	20147 (45.32)	262783 (60.08)
Retire	158901 (32.98)	18821 (42.34)	140080 (32.03)
Other	40010 (8.30)	5487 (12.34)	34523 (7.89)
College attendance (%)	160058 (33.22)	10864 (24.44)	149194 (34.11)
Ethnicity (%)			
White people	438226 (90.95)	40575 (91.27)	397651 (90.92)
Black people	2692 (0.56)	236 (0.53)	2456 (0.56)
Asian	18509 (3.84)	1423 (3.20)	17086 (3.91)
Other	22414 (4.65)	2221 (5.00)	20193 (4.62)
Physical activity (MET-mins)	2660.72 (2722.42)	2627.05 (2787.36)	2664.14 (2715.71)
Smoking status (%)			
Never	265278 (55.06)	21685 (48.78)	243593 (55.69)
Previous	165466 (34.34)	17111 (38.49)	148355 (33.92)
Current	51097 (10.60)	5659 (12.73)	45438 (10.39)
Drinking status (%)			
Never	20759 (4.31)	2591 (5.83)	18168 (4.15)
Previous	16544 (3.43)	2650 (5.96)	13894 (3.18)
Current	444538 (92.26)	39214 (88.21)	405324 (92.67)
Alcohol consumption frequency (%)			
Daily or almost daily	99307 (20.61)	7841 (17.64)	91466 (20.91)
3-4 Times a week	112651 (23.38)	8625 (19.40)	104026 (23.78)
1-2 Times a week	124879 (25.92)	11180 (25.15)	113699 (26.00)
1-3 Times a month	53451 (11.09)	5178 (11.65)	48273 (11.04)
Special occasions only	54226 (11.25)	6388 (14.37)	47838 (10.94)
None	37327 (7.75)	5243 (11.79)	32084 (7.34)
Healthy diet (fruit and vegetables)	382406 (79.36)	34436 (77.46)	347970 (79.56)
Healthy diet (fish)	216090 (44.85)	20200 (45.44)	195890 (44.79)
Healthy diet (processed meat)	192105 (39.87)	17817 (40.08)	174288 (39.85)
Healthy diet (red meat)	213427 (44.29)	19213 (43.22)	194214 (44.40)
Healthy diet (milk)	405794 (84.22)	36673 (82.49)	369121 (84.39)
Healthy diet (whole grains)	51999 (10.79)	4493 (10.11)	47506 (10.86)
Healthy diet (refined grains)	218476 (45.34)	20470 (46.05)	198006 (45.27)
Healthy diet (salt)	267597 (55.54)	22868 (51.44)	244729 (55.95)
Healthy diet (water)	52016 (10.80)	4997 (11.24)	47019 (10.75)
Social score	0.57 (0.67)	0.64 (0.70)	0.57 (0.67)
Social isolation (%)	44137 (9.16)	5023 (11.30)	39114 (8.94)
Overall health status (%)			
Excellent	81316 (16.88)	3371 (7.58)	77945 (17.82)
Good	283135 (58.76)	22222 (49.99)	260913 (59.65)
Fair	98172 (20.37)	14236 (32.02)	83936 (19.19)
Poor	19218 (3.99)	4626 (10.41)	14592 (3.34)
History of cancer (%)	36001 (7.47)	6560 (14.76)	29441 (6.73)
Vitamin supplement (%)	152993 (31.75)	15056 (33.87)	137937 (31.54)
CRP level (mg/L)	2.56 (4.30)	3.23 (5.18)	2.49 (4.20)

WHR, waist to hip ratio; BMI, body mass index; METS, metabolic equivalents task score; CRP, C-reactive protein.

### Gastrointestinal diseases with risk of endocrine diseases

**Figure 1:**
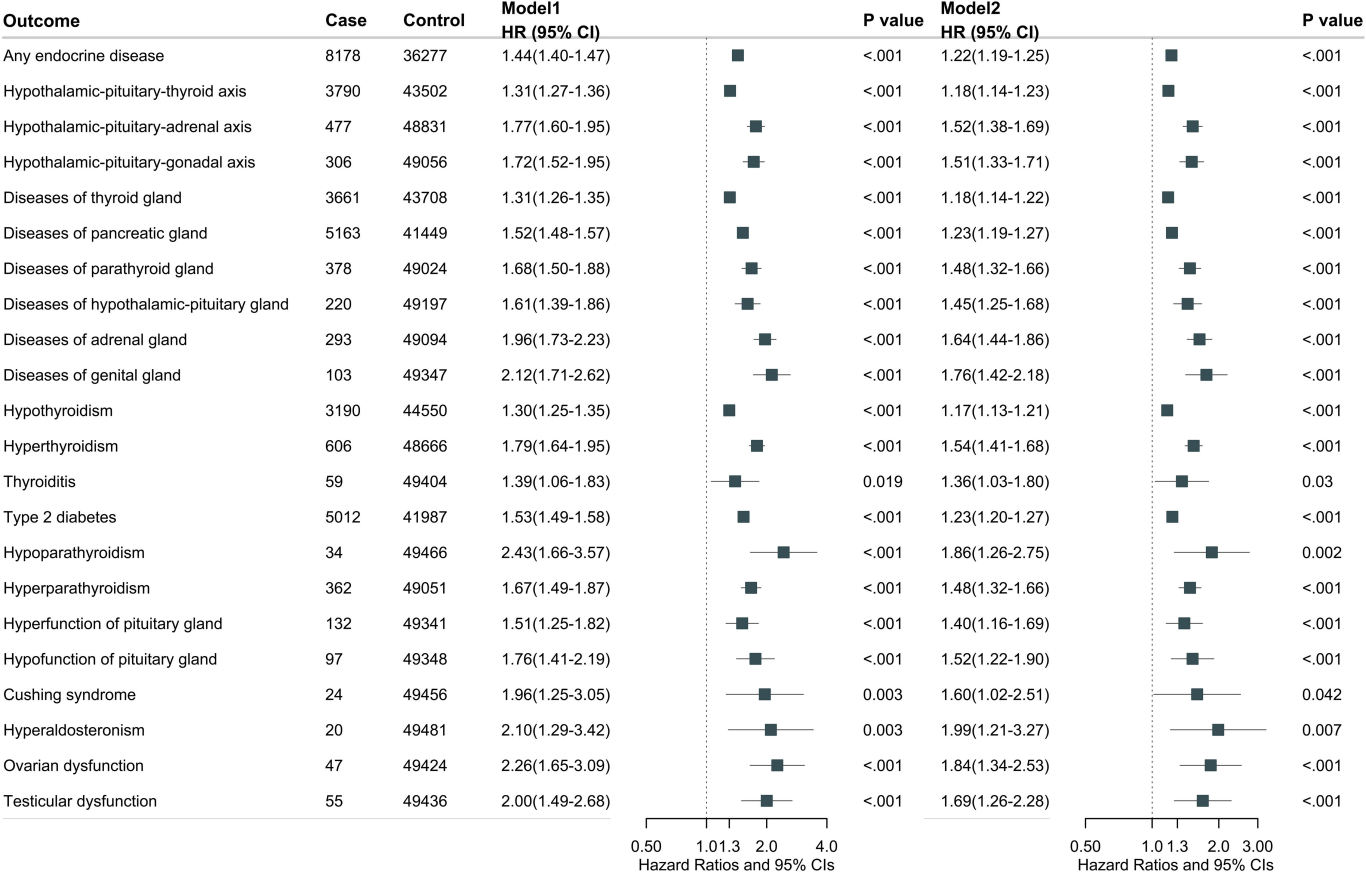
Overall gastrointestinal diseases associated with total and individual endocrine disease. Model 1 was adjusted for age at recruitment, sex, and ethnicity. Model 2 was further adjusted for BMI, Townsend deprivation index, employment, education, physical activity, smoking status, drinking status, adherence to a healthy diet, social isolation, and C-reactive protein.

During a median follow-up of 13.7 years (interquartile range 12.8, 14.5), we documented 8178 cases of endocrine diseases in participants with gastrointestinal diseases and 53190 cases in those without gastrointestinal diseases. Overall, gastrointestinal diseases were associated with total endocrine disease risk after multivariable adjustment (model 2: HR, 1.22; 95% CI, 1.19-1.25) (**Figure 1**). Further adjustment for medication use did not substantially alter the results (HR, 1.14; 95% CI, 1.11-1.16) (**Supplementary Table S5**). For individual endocrine diseases, participants with any gastrointestinal diseases had higher risks of hypothyroidism (HR, 1.17; 95% CI, 1.13-1.21), hyperthyroidism (HR, 1.54; 95% CI, 1.41-1.68), T2D (HR, 1.23; 95% CI, 1.20-1.27), hyperparathyroidism (HR, 1.48; 95% CI, 1.32-1.66), hyperpituitarism (HR, 1.40; 95% CI, 1.16-1.69), hypopituitarism (HR, 1.52; 95% CI, 1.22-1.90), ovarian dysfunction (HR, 1.84; 95% CI, 1.34-2.53), and testicular dysfunction (HR, 1.69; 95% CI, 1.26-2.28). Further adjustment for medication use did not substantially alter these results (**Supplementary Table S5**).

In addition, we assessed the effect of individual gastrointestinal disease on the risk of total endocrine diseases. Duodenal ulcer (HR, 1.21; 95% CI, 1.10-1.34), gastritis and duodenitis (HR, 1.20; 95% CI, 1.16-1.25), dyspepsia (HR, 1.16; 95% CI, 1.10-1.23), irritable bowel syndrome (HR, 1.31; 95% CI, 1.21-1.43), intestinal malabsorption (HR, 1.29; 95% CI, 1.12-1.50), and gastrointestinal hemorrhage (HR, 1.19; 95% CI, 1.10-1.29) were significantly associated with an increased risk of total endocrine diseases (**Supplementary Figure S2**).

### Endocrine diseases with risk of gastrointestinal diseases

**Figure 2:**
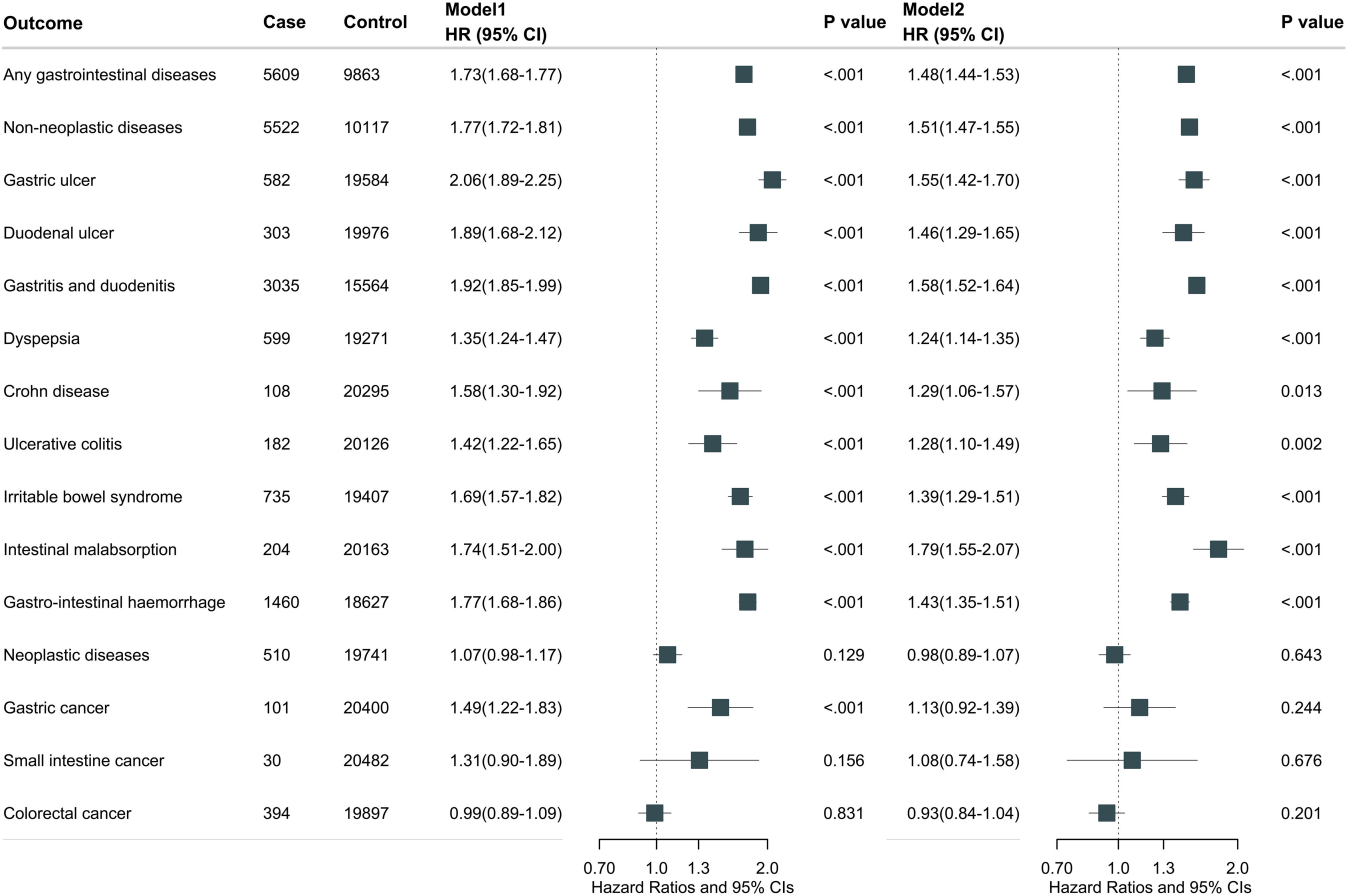
Overall endocrine diseases associated with total and individual gastrointestinal disease. Model 1 was adjusted for age at recruitment, sex, and ethnicity. Model 2 was further adjusted for BMI, Townsend deprivation index, employment, education, physical activity, smoking status, drinking status, adherence to a healthy diet, social isolation, and C-reactive protein.

During a median follow-up of 13.4 years (interquartile range 10.5, 14.3), we documented 5609 cases of gastrointestinal diseases in participants with endocrine diseases and 92369 cases in those without endocrine diseases. Overall, endocrine diseases were associated with total gastrointestinal disease risk after multivariable adjustment (model 2: HR, 1.48; 95% CI, 1.44-1.53) (**Figure 2**). Further adjustment for medication use did not substantially alter the results (HR, 1.34; 95% CI, 1.30-1.38) (**Supplementary Table S6**). For individual gastrointestinal diseases, participants with any endocrine diseases had higher risks of gastric ulcer (HR, 1.55; 95% CI, 1.42-1.70), duodenal ulcer (HR, 1.46; 95% CI, 1.29-1.65), gastritis and duodenitis (HR, 1.58; 95% CI, 1.52-1.64), dyspepsia (HR, 1.24; 95% CI, 1.14-1.35), irritable bowel syndrome (HR, 1.39; 95% CI, 1.29-1.51), intestinal malabsorption (HR, 1.79; 95% CI, 1.55-2.07), and gastrointestinal hemorrhage (HR, 1.43; 95% CI, 1.35-1.51). Further adjustment for medication use did not substantially alter these results (**Supplementary Table S6**).

In addition, we assessed the effect of individual endocrine disease on the risk of total gastrointestinal diseases. Hypothyroidism (HR, 1.46; 95% CI, 1.39-1.53), hyperthyroidism (HR, 1.29; 95% CI, 1.14-1.45), T2D (HR, 1.55; 95% CI, 1.49-1.51), hyperparathyroidism (HR, 1.77; 95% CI, 1.45-2.17), and hyperaldosteronism (HR, 2.31; 95% CI, 1.55-3.45) were significantly associated with an increased risk of total gastrointestinal diseases (**Supplementary Figure S3**).

### Bidirectional association between individual gastrointestinal diseases and individual endocrine diseases

**Figure 3:**
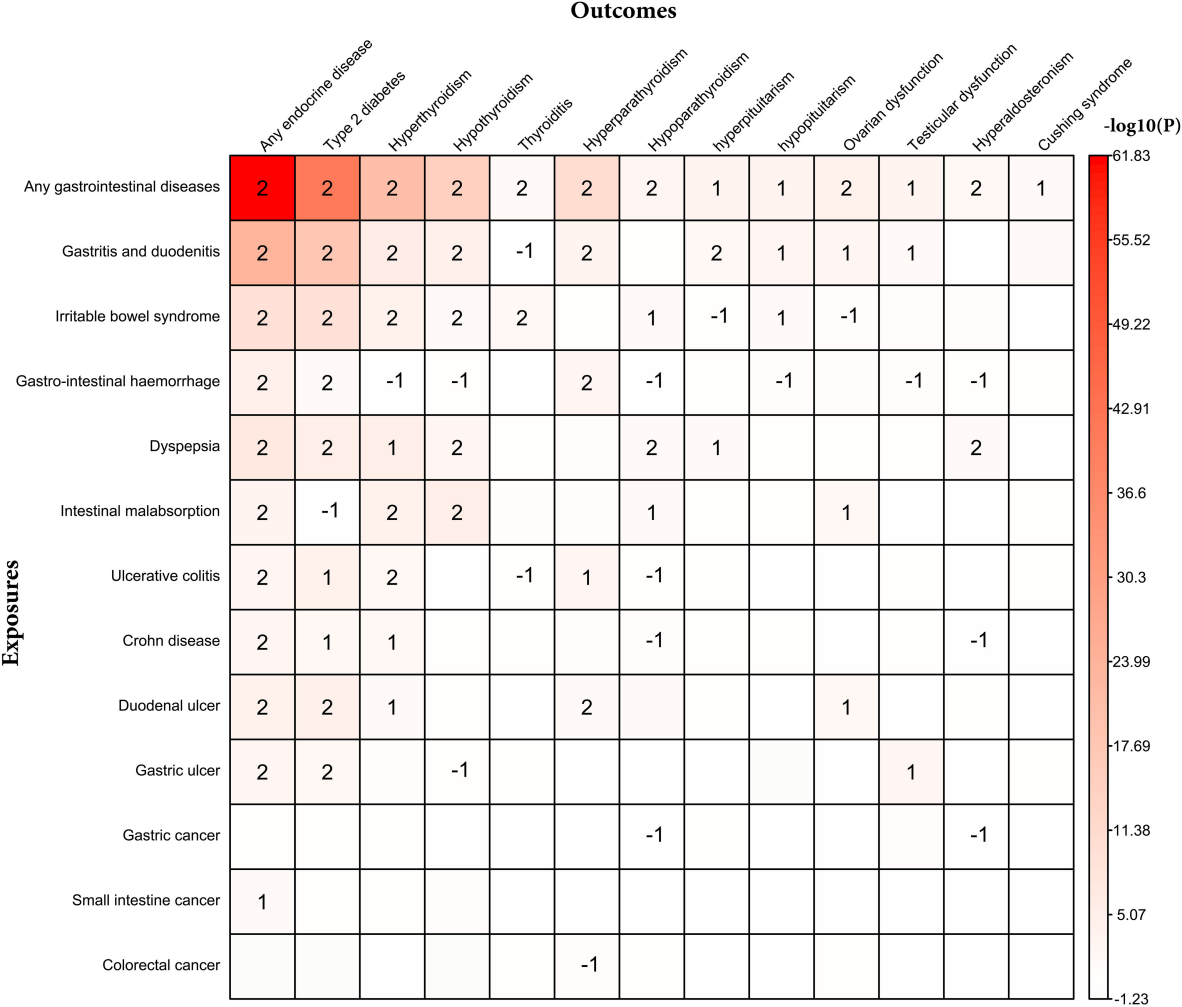
Bidirectional association between individual endocrine disease and individual gastrointestinal disease. 2 represents bidirectional association; 1 represents that individual gastrointestinal disease associated with individual endocrine disease; -1 represents that individual endocrine disease associated with individual gastrointestinal disease.

The site-specific analysis associating individual gastrointestinal diseases and individual endocrine diseases is shown in **Figure 3** and **Supplementary Tables S7**, **S8**. There is a bidirectional association between type 2 diabetes and six gastrointestinal diseases (gastritis and duodenitis, irritable bowel syndrome, gastrointestinal hemorrhage, dyspepsia, duodenal ulcer, and gastric ulcer); hyperthyroidism and four gastrointestinal diseases (gastritis and duodenitis, irritable bowel syndrome, malabsorption, and ulcerative colitis); hypothyroidism and four gastrointestinal diseases (gastritis and duodenitis, irritable bowel syndrome, malabsorption, and dyspepsia); hyperparathyroidism and three gastrointestinal diseases (gastritis and duodenitis, gastrointestinal hemorrhage, and duodenal ulcer).

### Gastrointestinal and endocrine comorbidity with risk of total and individual endocrine and gastrointestinal diseases respectively

**Table 2** shows the association between the number of gastrointestinal comorbidity and total and individual endocrine diseases. We observed a significant trend (P trend) for almost all endocrine diseases when subtyped by the number of gastrointestinal diseases, with the exception of thyroiditis, Cushing syndrome, and hyperaldosteronism. For most related endocrine diseases, participants with multiple gastrointestinal comorbidities (two or more) exhibited higher risk estimates compared to those with a single gastrointestinal disease. **Supplementary Table S9** shows the association between the number of endocrine comorbidity and total and individual gastrointestinal diseases. We observed a significant trend (P trend) for all non-neoplastic gastrointestinal diseases but not for neoplastic gastrointestinal diseases when subtyped by the number of endocrine diseases. For non-neoplastic diseases, participants with multiple endocrine comorbidities (two or more) exhibited higher risk estimates compared to those with a single endocrine disease.

**Table 2:** HR (95% CI) of the number of gastrointestinal comorbidities associated with total and individual endocrine diseases^$^.

Outcomes	Number of comorbidities	HR (95% CI)	P-value	HR (95% CI) for trend	P for trend
Any endocrine disease	1	1.19 (1.16-1.23)	<.001	1.14 (1.12-1.16)	<.001
	2	1.21 (1.14-1.29)	<.001		
	≥3	1.33 (1.16-1.54)	<.001		
Hypothalamic-pituitary-thyroid axis	1	1.17 (1.12-1.22)	<.001	1.10 (1.06-1.13)	<.001
	2	1.09 (0.99-1.20)	0.092		
	≥3	1.07 (0.84-1.36)	0.577		
Hypothalamic-pituitary-adrenal axis	1	1.43 (1.26-1.62)	<.001	1.29 (1.19-1.39)	<.001
	2	1.43 (1.11-1.85)	0.006		
	≥3	1.83 (1.10-3.05)	0.019		
Hypothalamic-pituitary-gonadal axis	1	1.37 (1.17-1.60)	<.001	1.28 (1.16-1.42)	<.001
	2	1.88 (1.42-2.50)	<.001		
	≥3	0.77 (0.29-2.05)	0.597		
Diseases of thyroid gland	1	1.16 (1.11-1.22)	<.001	1.09 (1.06-1.13)	<.001
	2	1.07 (0.97-1.18)	0.207		
	≥3	1.10 (0.86-1.39)	0.454		
Diseases of pancreatic gland	1	1.19 (1.15-1.23)	<.001	1.15 (1.12-1.18)	<.001
	2	1.26 (1.17-1.36)	<.001		
	≥3	1.50 (1.28-1.76)	<.001		
Diseases of parathyroid gland	1	1.38 (1.20-1.59)	<.001	1.28 (1.17-1.40)	<.001
	2	1.57 (1.18-2.08)	0.002		
	≥3	1.56 (0.81-3.01)	0.181		
Diseases of hypothalamic-pituitary gland	1	1.34 (1.12-1.61)	0.002	1.24 (1.10-1.39)	<.001
	2	1.73 (1.23-2.43)	0.001		
	≥3	0.50 (0.13-2.01)	0.331		
Diseases of adrenal gland	1	1.50 (1.27-1.76)	<.001	1.33 (1.20-1.47)	<.001
	2	1.29 (0.90-1.84)	0.164		
	≥3	2.77 (1.60-4.80)	<.001		
Diseases of genital gland	1	1.54 (1.17-2.04)	0.002	1.45 (1.22-1.72)	<.001
	2	2.35 (1.44-3.82)	<.001		
	≥3	1.44 (0.36-5.78)	0.608		
Hypothyroidism	1	1.15 (1.10-1.20)	<.001	1.09 (1.05-1.12)	<.001
	2	1.08 (0.97-1.20)	0.175		
	≥3	1.11 (0.86-1.43)	0.417		
Hyperthyroidism	1	1.52 (1.36-1.69)	<.001	1.32 (1.23-1.42)	<.001
	2	1.51 (1.19-1.91)	<.001		
	≥3	1.47 (0.85-2.53)	0.168		
Thyroiditis	1	1.10 (0.75-1.59)	0.63	1.16 (0.91-1.49)	0.236
	2	0.99 (0.41-2.40)	0.98		
	≥3	NA	0.025		
Type 2 diabetes	1	1.20 (1.15-1.24)	<.001	1.15 (1.12-1.18)	<.001
	2	1.22 (1.13-1.32)	<.001		
	≥3	1.45 (1.23-1.71)	<.001		
Hypoparathyroidism	1	1.82 (1.14-2.90)	0.013	1.43 (1.06-1.93)	0.018
	2	1.60 (0.59-4.36)	0.358		
	≥3	NA	0.511		
Hyperparathyroidism	1	1.38 (1.19-1.59)	<.001	1.28 (1.16-1.40)	<.001
	2	1.57 (1.18-2.10)	0.002		
	≥3	1.47 (0.73-2.94)	0.282		
hyperpituitarism	1	1.37 (1.08-1.72)	0.008	1.24 (1.06-1.44)	0.006
	2	1.69 (1.09-2.61)	0.019		
	≥3	0.41 (0.06-2.93)	0.376		
hypopituitarism	1	1.29 (0.96-1.71)	0.087	1.23 (1.02-1.47)	0.029
	2	1.77 (1.05-2.96)	0.031		
	≥3	0.59 (0.08-4.19)	0.597		
Cushing syndrome	1	0.93 (0.47-1.84)	0.828	1.20 (0.81-1.78)	0.36
	2	2.55 (1.03-6.30)	0.042		
	≥3	NA	0.99		
Hyperaldosteronism	1	1.96 (1.09-3.53)	0.025	1.16 (0.72-1.86)	0.539
	2	NA	0.993		
	≥3	NA	0.997		
Ovarian dysfunction	1	1.59 (1.04-2.42)	0.033	1.62 (1.25-2.11)	<.001
	2	3.06 (1.50-6.23)	0.002		
	≥3	NA	0.306		
Testicular dysfunction	1	1.54 (1.07-2.23)	0.022	1.35 (1.08-1.70)	0.009
	2	1.96 (1.01-3.83)	0.048		
	≥3	0.99 (0.14-7.07)	0.991		

^$^Cox model was adjusted for sex, age, BMI, Townsend deprivation index, employment, education, ethnicity, physical activity, smoking status, drinking status, adherence to a healthy diet, social isolation, and C-reactive protein.

HR, Hazard ratio; CI, Confidence interval; BMI, body mass index; NA, not available.

### Sensitivity analyses and stratified analyses

In the sensitivity analyses, when we excluded participants who had missing data on covariates, incident cases that occurred within the first years of follow-up, or participants with new-onset exposure diseases during follow-up, the results consistent with the main findings (**Supplementary Tables S10**-**S15**). Stratified analyses revealed that the main findings were not different by sex, age, smoking status, alcohol intake, BMI, and medication use (**Supplementary Tables S16**-**S19**).

## Discussions

In this population-based cohort study, we found that overall gastrointestinal disease was associated with an increased risk of incident total and individual endocrine diseases. Conversely, overall endocrine disease is associated with a higher risk of incident gastrointestinal non-neoplastic diseases. The study provided population-based evidence for the bidirectional association between gastrointestinal disease and endocrine diseases. Understanding this intricate and reciprocal association is essential for comprehensive patient care.

In our study, gastritis and duodenitis, irritable bowel syndrome, gastrointestinal hemorrhage, dyspepsia, duodenal ulcer, and gastric ulcer were associated with an increased risk of T2D. Conversely, T2D was linked to a higher risk of developing gastritis and duodenitis, irritable bowel syndrome, gastrointestinal hemorrhage, dyspepsia, duodenal ulcer, and gastric ulcer. These findings are partially consistent with prior studies. For example, a prospective cohort study in French found an association of chronic diarrhea or alternation diarrhea-constipation with a 15%-29% increased risk of T2D (17). Additionally, T2D was associated with a 58% higher risk of gastritis and duodenitis and a 56% higher risk of gastric ulcer (7). Previous studies have primarily focused on the unidirectional relationship between these diseases. To our knowledge, this study is the first comprehensive attempt to analyze the bidirectional association. Moreover, we reported for the first time the bidirectional association between gastrointestinal hemorrhage, dyspepsia, or duodenal ulcer, and T2D. Our findings also indicated that inflammatory bowel disease significantly increased the risk of T2D. Previous studies have shown conflicting results regarding the causal relationship between inflammatory bowel disease and the risk of T2D (18–21).

Several mechanisms might explain the bidirectional relationship between gastrointestinal diseases and T2D. Both conditions involve chronic low-grade inflammation, which exacerbates insulin resistance and adversely affects gastrointestinal health (22, 23). This deterioration is further compounded by immune system dysregulation (24, 25). Moreover, there is evidence suggesting that gut–brain interactions play a central role in chronic abdominal pain symptoms and gastrointestinal dysfunction (13), and metabolic disturbances (26). Additionally, the gut microbiota and its metabolites, such as short-chain fatty acids, are crucial for maintaining metabolic and gastrointestinal health. Dysbiosis and alterations in microbial metabolites can influence both insulin sensitivity and gastrointestinal inflammation (27, 28).

We observed a significantly strong association between gastritis and duodenitis, irritable bowel syndrome, dyspepsia, malabsorption, and ulcerative colitis and a higher overall risk of thyroid disorders (e.g., hyperthyroidism, hypothyroidism, and thyroiditis). And conversely, thyroid disorders also promote the development of gastritis and duodenitis, irritable bowel syndrome, dyspepsia, malabsorption, and ulcerative colitis. Previous studies have shown controversy regarding the causal relationship between inflammatory bowel disease and the risk of thyroid disorders. Some studies found that ulcerative colitis was a potential risk factor for hyperthyroidism (29), which was consistent with our findings. However, some studies showed that ulcerative colitis might be a protective factor for hyper- and hypothyroidism (12, 30, 31). Existing evidence suggests an increased risk of hyperthyroidism, hypothyroidism, and thyroiditis in patients with celiac disease (32–37), which was in line with our reports. Furthermore, it has been demonstrated that thyroid disorders also increase the risk of celiac disease (38–40). Regarding the mechanisms of these findings, the influence of the composition of the gut microbiota on the availability of essential micronutrients (e.g., iodine, iron, and copper) for the thyroid gland in gastrointestinal diseases drives the malfunctioning of the thyroid (41). Additionally, the compromised intestinal barrier and increased permeability in gastrointestinal diseases facilitate the passage of antigens. This process can either activate the immune system or cause antigens to cross-react with extraintestinal tissues (14).

In this study, a bidirectional relationship between gastritis and duodenitis, gastrointestinal hemorrhage, or duodenal ulcer and hyperparathyroidism, which was partially consistent with prior studies. For example, a transversal prospective study in Italy found an association of gastritis with a threefold increased risk of hyperparathyroidism, and hyperparathyroidism with a fourfold increased risk of gastritis (42). Previous case reports described the rare coexistence of gastric ulcer, duodenal ulcer, or gastrointestinal hemorrhage and hyperparathyroidism (43, 44). In this study, we found that ulcerative colitis significantly increased the risk of hyperparathyroidism. To our knowledge, previously only a case has reported that primary hyperparathyroidism occurred in a patient with ulcerative colitis (45). In this study, we found that malabsorption significantly increased the risk of hypoparathyroidism. However previous studies reported that celiac disease increased the development of primary hyperparathyroidism (10, 46). The increased risk of hyperparathyroidism in patients with gastrointestinal disease can be attributed to several interconnected mechanisms: malabsorption of calcium and vitamin D (47), acid-base disturbances (48), chronic inflammation (49), and hormonal interactions (50).

We identified several mechanisms, such as chronic inflammation and gut-brain axis involvement, that explain the bidirectional relationship between gastrointestinal and endocrine diseases. However, the potential clinical implications of these findings could be more thoroughly explored. Understanding these shared mechanisms could open avenues for developing integrated treatment strategies that address both systems simultaneously. For example, anti-inflammatory therapies that target chronic inflammation could benefit patients with both endocrine and gastrointestinal diseases, improving outcomes in conditions like T2D and irritable bowel syndrome. Additionally, gut-brain axis modulation through probiotics or dietary interventions may alleviate symptoms in both domains, especially in metabolic disorders and gastrointestinal dysfunctions.

Furthermore, the clinical relevance of this study lies in its potential to inform intervention strategies for patients already diagnosed with one condition. For instance, patients with T2D could be monitored more closely for gastrointestinal issues, and vice versa, with early interventions potentially reducing the risk of disease progression. Exploring the role of specific medications, such as gut-targeted drugs or anti-diabetic therapies like GLP-1 agonists, could also reveal how current treatments impact both disease areas, ultimately shaping public health guidelines and offering more personalized care pathways. This integrated approach could enhance disease prevention and improve patient quality of life across multiple health domains.

There are some limitations to the present study. First, considering that this is an observational study, we cannot interpret bidirectional association between gastrointestinal disease and endocrine diseases as causality. Although the time-dependent or joint model can assess time-dependent confounding, it does not explain the bidirectional association between the two groups of diseases. Due to the lack of repeated measurement data in the UK Biobank and suitable statistical methods for bidirectional associations in an observational setting, we used two separate Cox models based on two independent cohort designs rather than a single modified model. Second, the ascertainment of endocrine diseases and gastrointestinal diseases was only based on inpatient records, which may have led to some diagnoses made in the outpatient setting being missed. However, given the prospective design, any misclassification in the exposure status potentially renders our results null. Third, the study identified gastrointestinal disease and endocrine disease cases using ICD-10 codes from the UK Biobank. However, the ICD code may not be sufficient to detect early-stage cases and often results in the underdiagnosis of many diseases, potentially leading to misclassification. Fourth, although we included a wide range of identifiable covariates to adjust for the potential confounding factors, residual or unknown confounding could not be excluded. Fifth, the same as other studies conducted using UK Biobank, 85% of participants were White people, thus our results may limit generalizability. Finally, because of the nature of observational studies, reverse causality is still a possibility although the exclusion of participants with gastrointestinal disease or endocrine diseases diagnosed within the first year of follow-up in sensitivity analysis.

## Conclusions

In this cohort study, we observed an extensive bidirectional association in overall, specific, and number of comorbidities between gastrointestinal and endocrine diseases. Our findings offer population-based evidence of a bidirectional association between gastrointestinal and endocrine diseases. This knowledge could enhance intervention and treatment strategies for both conditions, thereby reducing the risk of developing one disease in patients already diagnosed with the other.

## Data Availability

The original contributions presented in the study are included in the article/**Supplementary Material**. Further inquiries can be directed to the corresponding author.
