# Supplementary material for: Dissecting the bidirectional associations between the progression of gastrointestinal and endocrine diseases
Source: Front Endocrinol (Lausanne). 2025 May 6;16:1538603. doi: 10.3389/fendo.2025.1538603 (PMC12088960; doi:10.3389/fendo.2025.1538603)
Supplement: Supplementary file 1 [file DataSheet1.docx]

Table S1 The list of gastrointestinal diseases and their ICD-10 codes

| **Gastrointestinal diseases** | **ICD-10 codes** |
| --- | --- |
| **Any gastrointestinal diseases** | K25-K31, K50-K52, K58-K59, K90-K92, C16-C20 |
| **Non-neoplastic diseases** | K25-K31, K50-K52, K58-K59, K90-K92 |
| Gastric ulcer | K25 |
| Duodenal ulcer | K26 |
| Gastritis and duodenitis | K29 |
| Dyspepsia | K30 |
| Crohn disease | K50 |
| Ulcerative colitis | K51 |
| Irritable bowel syndrome | K58 |
| Intestinal malabsorption | K90 |
| Gastro-intestinal haemorrhage | K920, K921, K922 |
| **Neoplastic diseases** | C16-C20 |
| Gastric cancer | C16 |
| Small intestine cancer | C17 |
| Colorectal cancer | C18-C20 |

Table S2 The list of endocrine diseases and their ICD-10 codes

| **Endocrine diseases** | **ICD-10 codes** |
| --- | --- |
| **Any** **endocrine diseases** | E00-E34 |
| **Hypothalamic-pituitary-thyroid axis** | E22, E23, E00-E07 |
| **Hypothalamic-pituitary-adrenal axis** | E22, E23, E24, E25, E26, E27 |
| **Hypothalamic-pituitary-gonadal axis** | E22, E23, E28, E29, E30 |
| **Diseases of thyroid gland** | E00-E07 |
| Hypothyroidism | E01, E02, E03 |
| Hyperthyroidism | E05 |
| Thyroiditis | E06 |
| **Diseases of pancreatic gland** | E10-E16 |
| Type 2 diabetes | E11 |
| **Diseases of parathyroid gland** | E20, E21 |
| Hypoparathyroidism | E20 |
| Hyperparathyroidism | E21 |
| **Diseases of pituitary gland** | E22, E23 |
| hyperpituitarism | E22 |
| hypopituitarism | E23 |
| **Diseases of adrenal gland** | E24, E25, E26, E27 |
| Cushing syndrome | E24 |
| Hyperaldosteronism | 426 |
| **Diseases of genital gland** | E28, E29, E30 |
| Ovarian dysfunction | 428 |
| Testicular dysfunction | E29 |

Table S3 Self-reported use of medications for gastrointestinal diseases and endocrine disease Treatment of at recruitment

| **Self-reported use of medications** | **Participants** |
| --- | --- |
| **Medications for gastrointestinal diseases** | 80452 |
| Drugs for acid related disorders (A02) | 59778 |
| Antacids (A02A) | 1049 |
| Drugs for peptic ulcer (A02B) | 59229 |
| Drugs for functional gastrointestinal disorders (A03) | 6329 |
| Drugs for functional gastrointestinal disorders (A03A) | 4140 |
| Belladonna and derivatives plain (A03B) | 625 |
| Antispasmodics in combination with psycholeptics (A03C) | 37 |
| Propulsives (A03F) | 1769 |
| Drugs for constipation (A06) | 8458 |
| Drugs for constipation (A06A) | 8458 |
| Antidiarrheals, intestinal anti-inflammatory/antiinfective agents (A07) | 18455 |
| Intestinal antiinfectives (A07A) | 89 |
| Antipropulsives (A07D) | 2033 |
| Intestinal anti-inflammatory agents (A07E) | 16625 |
| Digestives, incl. enzymes (A09) | 308 |
| Digestives, incl. enzymes (A09A) | 308 |
| **Medications for endocrine disease** | 50586 |
| Drugs used in diabetes (A10) | 19006 |
| Insulins and Analogues (A10A) | 5315 |
| Blood glucose lowering drugs, excl. insulins (A10B) | 16192 |
| Pituitary and Hypothalamic Hormones and Analogs (H01) | 299 |
| Anterior Pituitary Lobe Hormones and Analogues (H01A) | 117 |
| Posterior pituitary lobe hormones (H01B) | 167 |
| Hypothalamic Hormones (H01C) | 34 |
| Corticosteroids for systemic use (H02) | 5867 |
| Corticosteroids for Systemic Use, Plain (H02A) | 5867 |
| Thyroid Therapy (H03) | 28332 |
| Thyroid preparations (H03A) | 27962 |
| Antithyroid Preparations (H03B) | 434 |
| Pancreatic hormones (H04) | 14 |
| Glycogenolytic Hormones (H04A) | 14 |
| Calcium homeostasis (H05) | 20 |
| Anti-Parathyroid Agents (H05B) | 20 |


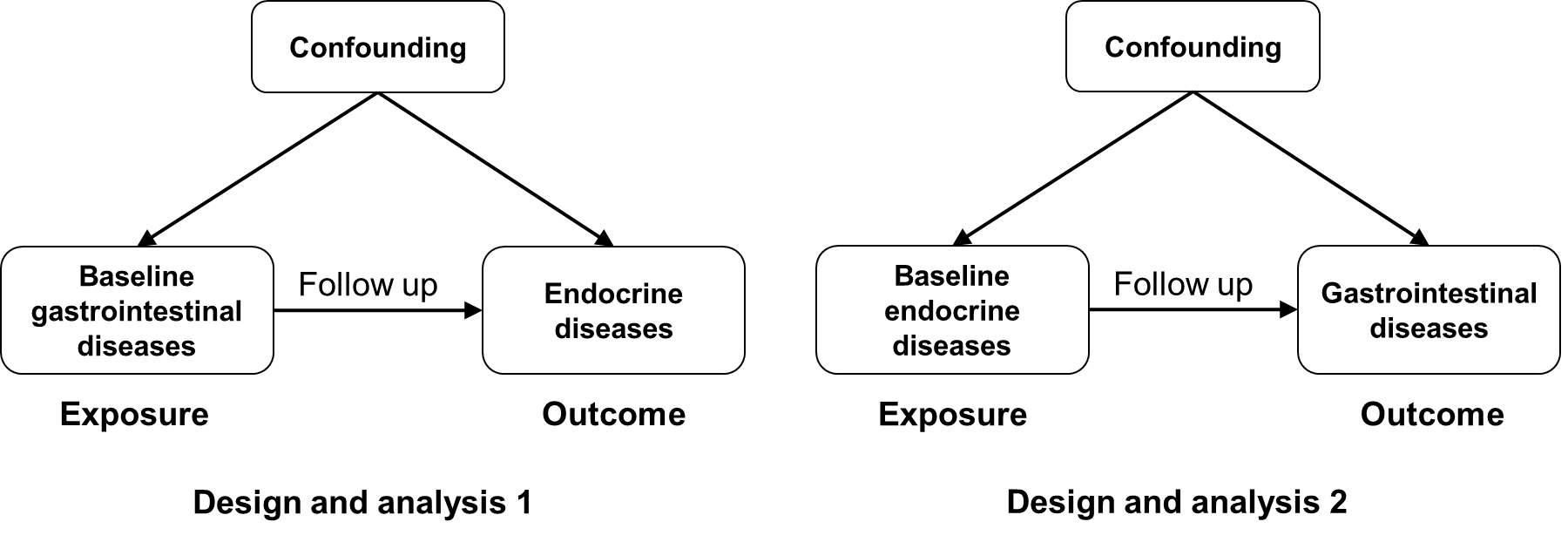


Figure S1. The design and analysis of this study.

Table S4 Baseline characteristics of study participants according to baseline endocrine diseases status

| **Baseline characteristics** | **Overall** | **Participants with endocrine diseases** | **Participants without endocrine diseases** |  |
| --- | --- | --- | --- | --- |
| Number of participants | 452858 | 15472 | 437386 |  |
| Age (years) | 56.30 (8.11) | 59.25 (7.49) | 56.20 (8.11) |  |
| Female (%) | 245297 (54.17) | 8929 (57.71) | 236368 (54.04) |  |
| WHR | 0.87 (0.09) | 0.90 (0.10) | 0.87 (0.09) |  |
| BMI (kg/m2) | 27.38 (4.77) | **30.25 (6.07)** | **27.28 (4.68)** |  |
| Townsend index | -1.35 (3.07) | **-0.69 (3.30)** | **-1.37 (3.06)** |  |
| Employment status (%) |  |  |  |  |
| Working | 268892 (59.38) | **6109 (39.48)** | **262783 (60.08)** |  |
| Retire | 147264 (32.52) | 7184 (46.43) | 140080 (32.03) |  |
| Other | 36702 (8.10) | 2179 (14.08) | 34523 (7.89) |  |
| College attendance (%) | 152855 (33.75) | **3661 (23.66)** | **149194 (34.11)** |  |
| Ethnicity (%) |  |  |  |  |
| White people | 411616 (90.89) | 13965 (90.26) | 397651 (90.92) |  |
| Black people | 2599 (0.57) | 143 (0.92) | 2456 (0.56) |  |
| Asian | 17597 (3.89) | 511 (3.30) | 17086 (3.91) |  |
| Other | 21046 (4.65) | 853 (5.51) | 20193 (4.62) |  |
| Physical activity (MET-mins) | 2653.91 (2713.54) | **2364.82 (2635.08)** | **2664.14 (2715.71)** |  |
| Smoking status (%) |  |  |  |  |
| Never | 251107 (55.45) | **7514 (48.57)** | **243593 (55.69)** |  |
| Previous | 154684 (34.16) | 6329 (40.91) | 148355 (33.92) |  |
| Current | 47067 (10.39) | 1629 (10.53) | 45438 (10.39) |  |
| Drinking status (%) |  |  |  |  |
| Never | 19434 (4.29) | **1266 (8.18)** | **18168 (4.15)** |  |
| Previous | 15018 (3.32) | 1124 (7.26) | 13894 (3.18) |  |
| Current | 418406 (92.39) | 13082 (84.55) | 405324 (92.67) |  |
| Alcohol consumption frequency (%) |  |  |  |  |
| Daily or almost daily | 93585 (20.67) | 2119 (13.70) | 91466 (20.91) |  |
| 3-4 times a week | 106434 (23.50) | 2408 (15.56) | 104026 (23.78) |  |
| 1-2 times a week | 117415 (25.93) | 3716 (24.02) | 113699 (26.00) |  |
| 1-3 times a month | 50209 (11.09) | 1936 (12.51) | 48273 (11.04) |  |
| Special occasions only | 50739 (11.20) | 2901 (18.75) | 47838 (10.94) |  |
| None | 34476 (7.61) | 2392 (15.46) | 32084 (7.34) |  |
| Healthy diet (fruit and vegetables) | 360449 (79.59) | 12479 (80.66) | 347970 (79.56) |  |
| Healthy diet (fish) | 202841 (44.79) | 6951 (44.93) | 195890 (44.79) |  |
| Healthy diet (processed meat) | 180333 (39.82) | 6045 (39.07) | 174288 (39.85) |  |
| Healthy diet (red meat) | 200736 (44.33) | 6522 (42.15) | 194214 (44.40) |  |
| Healthy diet (milk) | 382458 (84.45) | 13337 (86.20) | 369121 (84.39) |  |
| Healthy diet (whole grains) | 48968 (10.81) | 1462 (9.45) | 47506 (10.86) |  |
| Healthy diet (refined grains) | 205431 (45.36) | 7425 (47.99) | 198006 (45.27) |  |
| Healthy diet (salt) | 253151 (55.90) | 8422 (54.43) | 244729 (55.95) |  |
| Healthy diet (water) | 49007 (10.82) | 1988 (12.85) | 47019 (10.75) |  |
| Social score | 0.57 (0.67) | 0.69 (0.72) | 0.57 (0.67) |  |
| Social isolation (%) | 41056 (9.07) | **1942 (12.55)** | **39114 (8.94)** |  |
| Overall health status (%) |  |  |  |  |
| Excellent | 78673 (17.37) | 728 (4.71) | 77945 (17.82) |  |
| Good | 267251 (59.01) | 6338 (40.96) | 260913 (59.65) |  |
| Fair | 89895 (19.85) | 5959 (38.51) | 83936 (19.19) |  |
| Poor | 17039 (3.76) | 2447 (15.82) | 14592 (3.34) |  |
| History of cancer (%) | 31290 (6.91) | 1849 (11.95) | 29441 (6.73) |  |
| Vitamin supplement (%) | 142720 (31.52) | 4783 (30.91) | 137937 (31.54) |  |
| CRP level (mg/L) | 2.53 (4.25) | **3.53 (5.39)** | **2.49 (4.20)** |  |

Abbreviations: WHR: waist to hip ratio, BMI: body mass index, METS: metabolic equivalents task score, CRP: C-reactive protein.

Table S5 HR (95% CI) of any gastrointestinal diseases associated with total and individual endocrine disease further adjusted for medicine use^$^

| **Outcomes** | **HR (95% CI)** | **P value** |
| --- | --- | --- |
| **Any endocrine disease** | 1.14(1.11-1.16) | <.001 |
| **Hypothalamic-pituitary-thyroid axis** | 1.11(1.08-1.16) | <.001 |
| **Hypothalamic-pituitary-adrenal axis** | 1.24(1.12-1.38) | <.001 |
| **Hypothalamic-pituitary-gonadal axis** | 1.23(1.08-1.40) | 0.002 |
| **Diseases of thyroid gland** | 1.11(1.07-1.15) | <.001 |
| **Diseases of pancreatic gland** | 1.13(1.09-1.16) | <.001 |
| **Diseases of parathyroid gland** | 1.32(1.17-1.48) | <.001 |
| **Diseases of hypothalamic-pituitary gland** | 1.18(1.01-1.37) | 0.038 |
| **Diseases of adrenal gland** | 1.29(1.13-1.48) | <.001 |
| **Diseases of genital gland** | 1.41(1.12-1.77) | 0.003 |
| **Hypothyroidism** | 1.10(1.06-1.14) | <.001 |
| **Hyperthyroidism** | 1.41(1.29-1.55) | <.001 |
| **Thyroiditis** | 1.22(0.91-1.62) | 0.186 |
| **Type 2 diabetes** | 1.13(1.09-1.17) | <.001 |
| **Hypoparathyroidism** | 1.42(0.94-2.14) | 0.093 |
| **Hyperparathyroidism** | 1.33(1.18-1.50) | <.001 |
| **Hyperpituitarism** | 1.21(0.99-1.47) | 0.063 |
| **Hypopituitarism** | 1.10(0.88-1.39) | 0.407 |
| **Cushing syndrome** | 1.22(0.76-1.96) | 0.399 |
| **Hyperaldosteronism** | 1.81(1.08-3.06) | 0.026 |
| **Ovarian dysfunction** | 1.46(1.04-2.03) | 0.027 |
| **Testicular dysfunction** | 1.37(1.00-1.87) | 0.049 |

^$^Cox model was adjusted for sex, age, BMI, Townsend deprivation index, employment, education, ethnicity, physical activity, smoking status, drinking status, adherence to a healthy diet, social isolation, and C-reactive protein and medicine use.

Abbreviations: HR: Hazard ratio, CI: Confidence interval, BMI: body mass index.


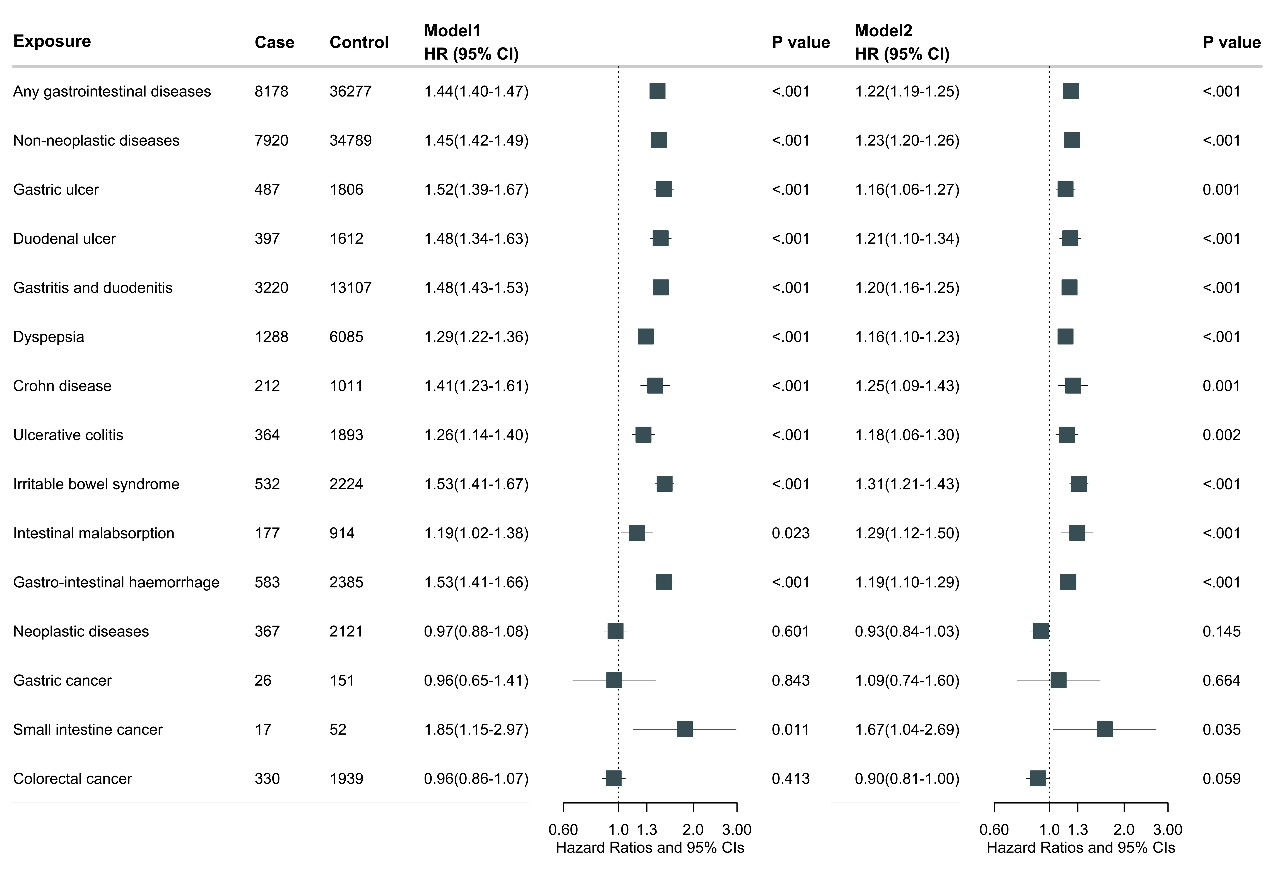


Figure S2. Individual gastrointestinal diseases associated with total endocrine diseases.

Model 1 was adjusted for age at recruitment, sex, and ethnicity. Model 2 was further adjusted for BMI, Townsend deprivation index, employment, education, physical activity, smoking status, drinking status, adherence to a healthy diet, social isolation, and C-reactive protein.

Table S6 HR (95% CI) of any endocrine disease associated with total and individual gastrointestinal diseases further adjusted for medicine use^$^

| **Outcomes** | **HR (95% CI)** | **P value** |
| --- | --- | --- |
| **Any gastrointestinal diseases** | 1.34(1.30-1.38) | <.001 |
| **Non-neoplastic diseases** | 1.35(1.31-1.40) | <.001 |
| **Gastric ulcer** | 1.38(1.25-1.53) | <.001 |
| **Duodenal ulcer** | 1.35(1.17-1.56) | <.001 |
| **Gastritis and duodenitis** | 1.39(1.33-1.45) | <.001 |
| **Dyspepsia** | 1.23(1.11-1.35) | <.001 |
| **Crohn disease** | 1.08(0.86-1.36) | 0.515 |
| **Ulcerative colitis** | 1.06(0.89-1.26) | 0.526 |
| **Irritable bowel syndrome** | 1.33(1.21-1.45) | <.001 |
| **Intestinal malabsorption** | 1.23(1.05-1.46) | 0.013 |
| **Gastro-intestinal haemorrhage** | 1.39(1.31-1.49) | <.001 |
| **Neoplastic diseases** | 0.98(0.88-1.08) | 0.673 |
| **Gastric cancer** | 1.08(0.85-1.37) | 0.536 |
| **Small intestine cancer** | 1.26(0.81-1.95) | 0.31 |
| **Colorectal cancer** | 0.94(0.84-1.06) | 0.323 |

^$^Cox model was adjusted for sex, age, BMI, Townsend deprivation index, employment, education, ethnicity, physical activity, smoking status, drinking status, adherence to a healthy diet, social isolation, and C-reactive protein and medicine use.

Abbreviations: HR: Hazard ratio, CI: Confidence interval, BMI: body mass index.


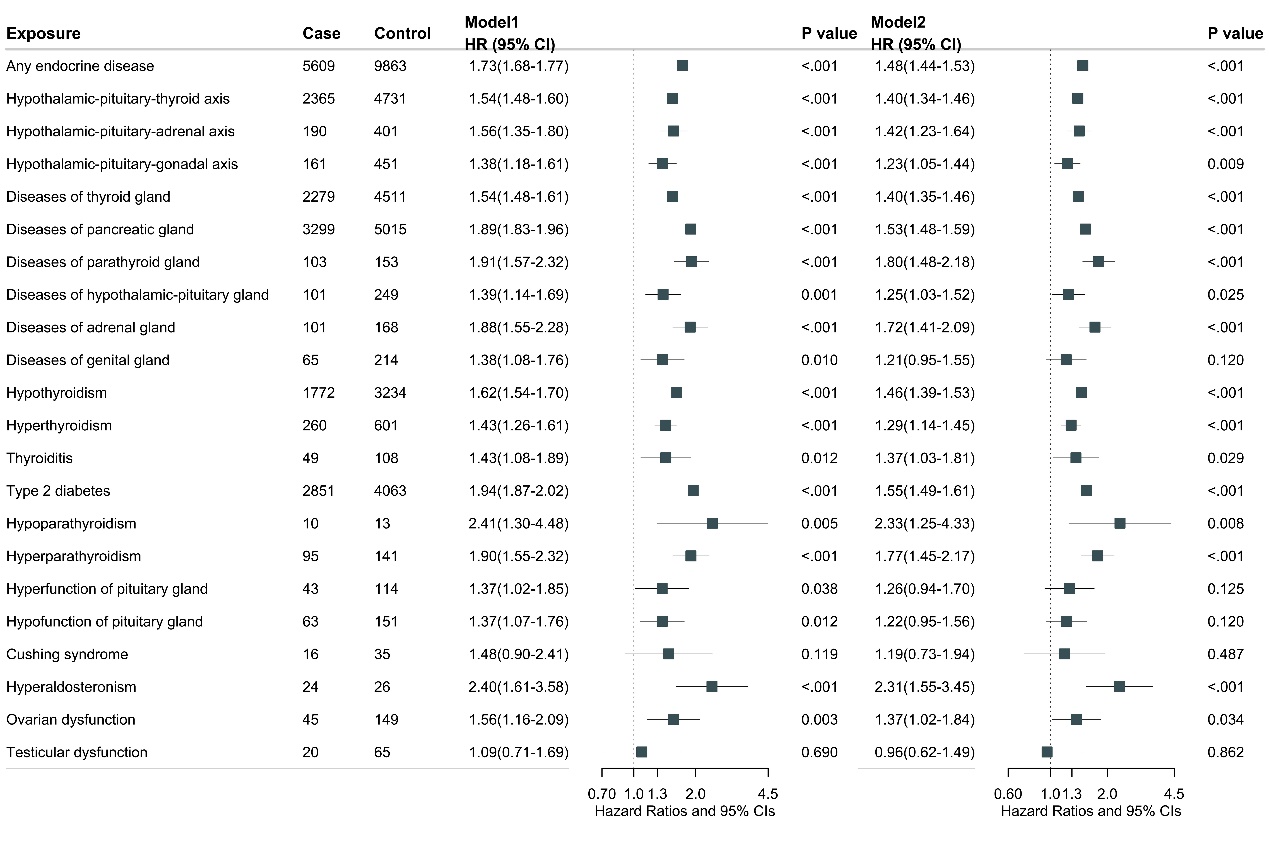


Figure S3. Individual endocrine diseases associated with total gastrointestinal diseases.

Model 1 was adjusted for age at recruitment, sex, and ethnicity. Model 2 was further adjusted for BMI, Townsend deprivation index, employment, education, physical activity, smoking status, drinking status, adherence to a healthy diet, social isolation, and C-reactive protein.

Table S7 HR (95% CI) and event numbers of individual gastrointestinal diseases associated with individual endocrine disease^$^

|  | **Hypothyroidism** | |  | **Hyperthyroidism** | |  | **Thyroiditis** |  |  |
| --- | --- | --- | --- | --- | --- | --- | --- | --- | --- |
|  | **Case/control** | **HR (95% CI)** | **P value** | **Case/control** | **HR (95% CI)** | **P value** | **Case/control** | **HR (95% CI)** | **P value** |
| **Gastric ulcer** | 164/2387 | 1.03(0.89-1.21) | 0.664 | 31/2609 | 1.26(0.88-1.79) | 0.205 | 4/2647 | 1.80(0.67-4.82) | 0.245 |
| **Duodenal ulcer** | 117/2069 | 1.08(0.90-1.29) | 0.43 | 27/2219 | 1.56(1.07-2.29) | **0.021** | 1/2254 | 0.66(0.09-4.69) | 0.676 |
| **Gastritis and duodenitis** | 1172/16418 | 1.14(1.07-1.21) | **<.001** | 218/17952 | 1.40(1.22-1.61) | **<.001** | 15/18226 | 0.95(0.57-1.60) | 0.848 |
| **Dyspepsia** | 545/7221 | 1.15(1.06-1.25) | **0.001** | 109/7893 | 1.56(1.29-1.89) | **<.001** | 10/8012 | 1.28(0.68-2.39) | 0.448 |
| **Crohn disease** | 72/1239 | 1.01(0.80-1.27) | 0.961 | 20/1321 | 1.80(1.16-2.80) | **0.009** | 2/1346 | 1.60(0.40-6.43) | 0.508 |
| **Ulcerative colitis** | 111/2311 | 0.91(0.75-1.09) | 0.297 | 29/2440 | 1.61(1.11-2.32) | **0.011** | 3/2472 | 1.50(0.48-4.67) | 0.486 |
| **Irritable bowel syndrome** | 228/2734 | 1.17(1.03-1.33) | **0.02** | 53/3069 | 1.75(1.34-2.30) | **<.001** | 9/3130 | 2.56(1.32-4.97) | **0.005** |
| **Intestinal malabsorption** | 103/1063 | 1.55(1.27-1.88) | **<.001** | 23/1215 | 2.24(1.49-3.38) | **<.001** | 3/1244 | 2.32(0.75-7.24) | 0.146 |
| **Gastro-intestinal haemorrhage** | 191/3084 | 1.08(0.93-1.24) | 0.303 | 27/3355 | 0.94(0.64-1.37) | 0.747 | 0/3407 | NA | 0.982 |
| **Gastric cancer** | 8/191 | 0.82(0.41-1.64) | 0.579 | 3/202 | 1.87(0.60-5.81) | 0.278 | 0/204 | NA | 0.985 |
| **Small intestine cancer** | 7/75 | 1.68(0.80-3.53) | 0.168 | 1/85 | NA | 0.691 | 0/85 | NA | 0.99 |
| **Colorectal cancer** | 113/2327 | 0.85(0.70-1.02) | 0.078 | 16/2486 | 0.81(0.50-1.33) | 0.402 | 2/2504 | 1.04(0.26-4.17) | 0.959 |

|  | **Type 2 diabetes** | |  | **Hypoparathyroidism** | |  | **Hyperparathyroidism** | | |
| --- | --- | --- | --- | --- | --- | --- | --- | --- | --- |
|  | **Case/control** | **HR (95% CI)** | **P value** | **Case/control** | **HR (95% CI)** | **P value** | **Case/control** | **HR (95% CI)** | **P value** |
| **Gastric ulcer** | 334/2113 | 1.16(1.04-1.29) | **0.007** | 0/2656 | NA | 0.99 | 14/2636 | 0.90(0.53-1.52) | 0.681 |
| **Duodenal ulcer** | 291/1824 | 1.28(1.14-1.44) | **<.001** | 3/2256 | 3.14(1.00-9.92) | 0.051 | 18/2235 | 1.69(1.06-2.70) | **0.026** |
| **Gastritis and duodenitis** | 2059/15165 | 1.23(1.17-1.28) | **<.001** | 11/18242 | 1.41(0.76-2.62) | 0.28 | 135/18093 | 1.39(1.16-1.66) | **<.001** |
| **Dyspepsia** | 744/6999 | 1.19(1.11-1.28) | **<.001** | 7/8025 | 2.22(1.04-4.76) | **0.04** | 52/7963 | 1.20(0.91-1.58) | 0.2 |
| **Crohn disease** | 125/1162 | 1.27(1.06-1.51) | **0.008** | 2/1346 | NA | 0.091 | 11/1334 | 1.72(0.95-3.11) | 0.075 |
| **Ulcerative colitis** | 238/2112 | 1.30(1.14-1.47) | **<.001** | 1/2477 | 1.06(0.15-7.57) | 0.955 | 22/2450 | 2.02(1.33-3.08) | **0.001** |
| **Irritable bowel syndrome** | 314/2673 | 1.43(1.28-1.60) | **<.001** | 4/3138 | 2.80(1.03-7.60) | 0.044 | 21/3115 | 1.17(0.76-1.80) | 0.476 |
| **Intestinal malabsorption** | 72/1141 | 0.95(0.75-1.19) | 0.638 | 2/1250 | NA | **0.045** | 10/1239 | 1.56(0.84-2.90) | 0.164 |
| **Gastro-intestinal haemorrhage** | 394/2767 | 1.13(1.02-1.24) | **0.019** | 1/3408 | 0.60(0.08-4.31) | 0.613 | 31/3372 | 1.81(1.27-2.58) | **0.001** |
| **Gastric cancer** | 16/168 | 1.19(0.73-1.94) | 0.487 | 0/205 | NA | 0.991 | 0/205 | NA | 0.967 |
| **Small intestine cancer** | 10/72 | 1.21(0.65-2.24) | 0.551 | 0/86 | NA | 0.992 | 0/85 | NA | 0.978 |
| **Colorectal cancer** | 206/2153 | 0.89(0.78-1.03) | 0.111 | 1/2506 | 1.00(0.14-7.18) | 0.998 | 19/2485 | 1.48(0.94-2.33) | 0.089 |

|  | **Hyperpituitarism** | | | **Hypopituitarism** | | | **Cushing syndrome** | |  |
| --- | --- | --- | --- | --- | --- | --- | --- | --- | --- |
|  | **Case/control** | **HR (95% CI)** | **P value** | **Case/control** | **HR (95% CI)** | **P value** | **Case/control** | **HR (95% CI)** | **P value** |
| **Gastric ulcer** | 6/2648 | 0.96(0.43-2.15) | 0.928 | 1/2653 | 0.23(0.03-1.63) | 0.141 | 2/2653 | 2.05(0.50-8.33) | 0.315 |
| **Duodenal ulcer** | 6/2253 | 1.15(0.52-2.58) | 0.728 | 3/2249 | 0.83(0.27-2.57) | 0.741 | 0/2257 | NA | 0.993 |
| **Gastritis and duodenitis** | 55/18183 | 1.45(1.10-1.91) | **0.008** | 42/18195 | 1.62(1.18-2.22) | **0.003** | 11/18240 | 1.84(0.99-3.44) | 0.056 |
| **Dyspepsia** | 24/8003 | 1.56(1.04-2.34) | **0.033** | 11/8015 | 1.08(0.59-1.96) | 0.803 | 2/8027 | 0.77(0.19-3.10) | 0.709 |
| **Crohn disease** | 3/1346 | 1.07(0.34-3.32) | 0.91 | 3/1346 | 1.62(0.52-5.04) | 0.406 | 0/1348 | NA | 0.992 |
| **Ulcerative colitis** | 6/2472 | 1.27(0.57-2.83) | 0.562 | 3/2473 | 0.88(0.28-2.74) | 0.827 | 1/2476 | NA | 0.678 |
| **Irritable bowel syndrome** | 7/3134 | 1.28(0.61-2.69) | 0.523 | 8/3132 | 2.11(1.05-4.25) | 0.037 | 0/3136 | NA | 0.992 |
| **Intestinal malabsorption** | 4/1248 | 1.58(0.59-4.23) | 0.361 | 2/1248 | 1.38(0.34-5.54) | 0.649 | 1/1250 | NA | 0.301 |
| **Gastro-intestinal haemorrhage** | 9/3400 | 1.20(0.62-2.32) | 0.589 | 10/3391 | 1.78(0.95-3.34) | 0.071 | 2/3403 | 1.71(0.42-6.97) | 0.451 |
| **Gastric cancer** | 1/204 | NA | 0.66 | 0/205 | NA | 0.982 | 0/205 | NA | 0.993 |
| **Small intestine cancer** | 0/86 | NA | 0.986 | 0/86 | NA | 0.988 | 0/86 | NA | 0.994 |
| **Colorectal cancer** | 5/2501 | 0.81(0.34-1.96) | 0.641 | 2/2504 | 0.53(0.13-2.14) | 0.374 | 0/2505 | NA | 0.99 |

|  | **Hyperaldosteronism** | |  | **Ovarian dysfunction** | |  | **Testicular dysfunction** | | |
| --- | --- | --- | --- | --- | --- | --- | --- | --- | --- |
|  | **Case/control** | **HR (95% CI)** | **P value** | **Case/control** | **HR (95% CI)** | **P value** | **Case/control** | **HR (95% CI)** | **P value** |
| **Gastric ulcer** | 0/2654 | NA | 0.992 | 1/2653 | 0.95(0.13-6.76) | 0.956 | 8/2647 | 3.21(1.58-6.51) | **0.001** |
| **Duodenal ulcer** | 1/2258 | NA | 0.615 | 3/2255 | NA | **0.011** | 1/2256 | 0.40(0.06-2.83) | 0.356 |
| **Gastritis and duodenitis** | 4/18248 | 0.90(0.33-2.46) | 0.839 | 18/18228 | 2.22(1.37-3.60) | **0.001** | 23/18227 | 1.61(1.05-2.47) | **0.03** |
| **Dyspepsia** | 5/8027 | 3.09(1.25-7.61) | **0.014** | 6/8023 | 1.31(0.58-2.95) | 0.513 | 6/8025 | 1.34(0.60-3.01) | 0.476 |
| **Crohn disease** | 0/1347 | NA | 0.992 | 0/1348 | NA | 0.987 | 2/1347 | 1.95(0.48-7.86) | 0.346 |
| **Ulcerative colitis** | 0/2478 | NA | 0.993 | 1/2475 | 0.85(0.12-6.08) | 0.873 | 2/2476 | 1.02(0.25-4.09) | 0.979 |
| **Irritable bowel syndrome** | 2/3140 | NA | 0.094 | 6/3130 | 2.04(0.91-4.60) | 0.085 | 3/3139 | 2.36(0.76-7.37) | 0.14 |
| **Intestinal malabsorption** | 0/1253 | NA | 0.992 | 3/1247 | NA | **0.013** | 0/1253 | NA | 0.986 |
| **Gastro-intestinal haemorrhage** | 1/3407 | 1.09(0.15-7.86) | 0.932 | 4/3405 | 1.95(0.72-5.26) | 0.19 | 7/3400 | 1.93(0.91-4.10) | 0.087 |
| **Gastric cancer** | 0/205 | NA | 0.992 | 0/205 | NA | 0.992 | 1/204 | NA | 0.07 |
| **Small intestine cancer** | 0/86 | NA | 0.995 | 0/86 | NA | 0.991 | 0/86 | NA | 0.991 |
| **Colorectal cancer** | 0/2507 | NA | 0.993 | 1/2505 | NA | 0.642 | 1/2506 | 0.44(0.06-3.13) | 0.411 |

^$^Cox model was adjusted for sex, age, BMI, Townsend deprivation index, employment, education, ethnicity, physical activity, smoking status, drinking status, adherence to a healthy diet, social isolation, and C-reactive protein.

Abbreviations: HR: Hazard ratio, CI: Confidence interval, BMI: body mass index.

Table S8 HR (95% CI) and event numbers of individual endocrine disease associated with individual gastrointestinal diseases^$^

|  | **Gastric ulcer** | |  | **Duodenal ulcer** | |  | **Gastritis and duodenitis** | | |
| --- | --- | --- | --- | --- | --- | --- | --- | --- | --- |
|  | **Case/control** | **HR (95% CI)** | **P value** | **Case/control** | **HR (95% CI)** | **P value** | **Case/control** | **HR (95% CI)** | **P value** |
| **Hypothyroidism** | 179/6494 | 1.56(1.35-1.82) | **<.001** | 65/6640 | 1.24(0.97-1.59) | 0.084 | 959/5152 | 1.54(1.44-1.64) | **<.001** |
| **Hyperthyroidism** | 23/1062 | 1.33(0.88-2.00) | 0.175 | 6/1082 | 0.71(0.32-1.59) | 0.407 | 128/886 | 1.29(1.08-1.53) | 0.004 |
| **Thyroiditis** | 5/196 | 1.83(0.76-4.39) | 0.178 | 2/200 | 1.74(0.43-6.95) | 0.435 | 26/164 | 1.53(1.04-2.24) | 0.031 |
| **Type 2 diabetes** | 325/8893 | 1.55(1.38-1.74) | **<.001** | 208/9075 | 1.67(1.44-1.93) | **<.001** | 1627/6767 | 1.67(1.59-1.76) | **<.001** |
| **Hypoparathyroidism** | 2/33 | NA | 0.073 | 0/35 | NA | 0.973 | 6/25 | 2.17(0.98-4.84) | 0.057 |
| **Hyperparathyroidism** | 4/325 | 0.70(0.26-1.86) | 0.472 | 7/322 | 2.48(1.18-5.20) | 0.017 | 51/255 | 1.64(1.25-2.16) | **<.001** |
| **Hyperpituitarism** | 4/190 | 1.34(0.50-3.57) | 0.558 | 3/193 | 1.89(0.61-5.87) | 0.27 | 30/147 | 1.92(1.34-2.74) | **<.001** |
| **Hypopituitarism** | 3/276 | 0.60(0.19-1.86) | 0.378 | 0/274 | NA | 0.966 | 29/232 | 1.08(0.75-1.56) | 0.671 |
| **Cushing syndrome** | 2/80 | 1.26(0.32-5.04) | 0.743 | 0/81 | NA | 0.971 | 11/66 | 1.30(0.72-2.34) | 0.388 |
| **Hyperaldosteronism** | 2/57 | 1.95(0.49-7.79) | 0.346 | 2/59 | NA | 0.073 | 8/48 | 1.46(0.73-2.92) | 0.286 |
| **Ovarian dysfunction** | 1/232 | 0.39(0.05-2.74) | 0.341 | 2/232 | 2.27(0.57-9.08) | 0.248 | 22/202 | 1.36(0.89-2.06) | 0.154 |
| **Testicular dysfunction** | 2/103 | 0.98(0.24-3.91) | 0.974 | 1/103 | 0.69(0.10-4.92) | 0.714 | 6/93 | 0.57(0.26-1.28) | 0.174 |

|  | **Dyspepsia** | |  | **Crohn disease** | |  | **Ulcerative colitis** | |  |
| --- | --- | --- | --- | --- | --- | --- | --- | --- | --- |
|  | **Case/control** | **HR (95% CI)** | **P value** | **Case/control** | **HR (95% CI)** | **P value** | **Case/control** | **HR (95% CI)** | **P value** |
| **Hypothyroidism** | 220/6292 | 1.26(1.11-1.45) | **<.001** | 38/6702 | 1.38(0.99-1.90) | 0.054 | 54/6668 | 1.22(0.93-1.59) | 0.158 |
| **Hyperthyroidism** | 27/1044 | 1.01(0.69-1.47) | 0.979 | 6/1087 | 1.29(0.58-2.88) | 0.535 | 15/1077 | 2.06(1.24-3.42) | 0.005 |
| **Thyroiditis** | 7/189 | 1.41(0.67-2.97) | 0.36 | 1/204 | 1.28(0.18-9.11) | 0.804 | 4/199 | 3.31(1.24-8.82) | 0.017 |
| **Type 2 diabetes** | 275/8863 | 1.25(1.10-1.41) | **<.001** | 53/9312 | 1.28(0.97-1.70) | 0.084 | 87/9212 | 1.20(0.97-1.50) | 0.098 |
| **Hypoparathyroidism** | 3/32 | NA | 0.023 | 2/32 | NA | **<.001** | 2/33 | NA | 0.002 |
| **Hyperparathyroidism** | 11/307 | 1.34(0.74-2.43) | 0.328 | 2/329 | 1.49(0.37-5.97) | 0.572 | 3/326 | 1.34(0.43-4.15) | 0.614 |
| **Hyperpituitarism** | 5/186 | 1.16(0.48-2.78) | 0.746 | 1/195 | 1.19(0.17-8.47) | 0.861 | 1/195 | 0.76(0.11-5.39) | 0.783 |
| **Hypopituitarism** | 4/271 | 0.62(0.23-1.66) | 0.342 | 1/280 | 0.85(0.12-6.07) | 0.875 | 2/277 | 1.00(0.25-4.01) | 0.997 |
| **Cushing syndrome** | 3/77 | 1.42(0.46-4.41) | 0.542 | 1/81 | NA | 0.348 | 0/82 | NA | 0.961 |
| **Hyperaldosteronism** | 6/55 | NA | **<.001** | 3/56 | NA | **<.001** | 0/61 | NA | 0.967 |
| **Ovarian dysfunction** | 8/224 | 1.67(0.83-3.34) | 0.148 | 1/233 | 1.07(0.15-7.60) | 0.948 | 0/233 | NA | 0.972 |
| **Testicular dysfunction** | 3/102 | 1.53(0.49-4.75) | 0.461 | 0/106 | NA | 0.972 | 0/106 | NA | 0.97 |

|  | **Irritable bowel syndrome** | | | **Intestinal malabsorption** | | | **Gastro-intestinal haemorrhage** | | |
| --- | --- | --- | --- | --- | --- | --- | --- | --- | --- |
|  | **Case/control** | **HR (95% CI)** | **P value** | **Case/control** | **HR (95% CI)** | **P value** | **Case/control** | **HR (95% CI)** | **P value** |
| **Hypothyroidism** | 331/6266 | 1.66(1.49-1.86) | **<.001** | 83/6608 | 1.94(1.56-2.42) | **<.001** | 440/6203 | 1.44(1.30-1.58) | **<.001** |
| **Hyperthyroidism** | 43/1037 | 1.38(1.02-1.86) | 0.036 | 18/1068 | 2.60(1.64-4.14) | **<.001** | 67/1006 | 1.40(1.10-1.78) | 0.006 |
| **Thyroiditis** | 12/190 | 2.01(1.14-3.54) | 0.016 | 2/198 | 1.49(0.37-5.95) | 0.575 | 13/190 | 1.62(0.94-2.80) | 0.08 |
| **Type 2 diabetes** | 290/8981 | 1.25(1.11-1.41) | **<.001** | 69/9318 | 1.40(1.09-1.78) | 0.007 | 793/8385 | 1.44(1.34-1.55) | **<.001** |
| **Hypoparathyroidism** | 0/34 | NA | 0.952 | 1/33 | NA | 0.128 | 5/29 | 3.49(1.45-8.40) | 0.005 |
| **Hyperparathyroidism** | 10/318 | 1.06(0.57-1.96) | 0.862 | 2/329 | 0.90(0.22-3.59) | 0.879 | 32/296 | 2.06(1.45-2.91) | **<.001** |
| **Hyperpituitarism** | 10/184 | 1.97(1.06-3.66) | 0.032 | 1/194 | 0.87(0.12-6.21) | 0.894 | 12/183 | 1.36(0.77-2.40) | 0.286 |
| **Hypopituitarism** | 11/267 | 1.60(0.89-2.90) | 0.118 | 2/276 | 1.23(0.31-4.93) | 0.767 | 27/245 | 1.96(1.34-2.85) | **<.001** |
| **Cushing syndrome** | 2/74 | 0.77(0.19-3.08) | 0.713 | 1/80 | NA | 0.517 | 5/73 | 1.23(0.51-2.96) | 0.643 |
| **Hyperaldosteronism** | 0/60 | NA | 0.938 | 1/60 | NA | 0.272 | 7/52 | 2.51(1.19-5.26) | 0.015 |
| **Ovarian dysfunction** | 13/215 | 1.76(1.02-3.03) | 0.042 | 1/231 | 0.76(0.11-5.38) | 0.781 | 7/227 | 0.86(0.41-1.80) | 0.685 |
| **Testicular dysfunction** | 1/104 | 0.62(0.09-4.38) | 0.629 | 0/106 | NA | 0.972 | 12/91 | 2.11(1.20-3.71) | 0.01 |

|  | **Gastric cancer** | |  | **Small intestine cancer** | | | **Colorectal cancer** | |  |
| --- | --- | --- | --- | --- | --- | --- | --- | --- | --- |
|  | **Case/control** | **HR (95% CI)** | **P value** | **Case/control** | **HR (95% CI)** | **P value** | **Case/control** | **HR (95% CI)** | **P value** |
| **Hypothyroidism** | 20/6752 | 0.98(0.63-1.53) | 0.933 | 9/6765 | 1.13(0.58-2.20) | 0.714 | 103/6608 | 0.83(0.68-1.01) | 0.059 |
| **Hyperthyroidism** | 3/1098 | 0.96(0.31-2.97) | 0.94 | 2/1099 | 1.70(0.42-6.84) | 0.453 | 15/1081 | 0.82(0.49-1.36) | 0.434 |
| **Thyroiditis** | 0/205 | NA | 0.974 | 1/204 | NA | 0.093 | 4/201 | 1.30(0.49-3.45) | 0.604 |
| **Type 2 diabetes** | 67/9339 | 1.18(0.92-1.52) | 0.195 | 17/9406 | 1.09(0.66-1.79) | 0.731 | 214/9065 | 0.95(0.82-1.09) | 0.429 |
| **Hypoparathyroidism** | 1/34 | NA | 0.018 | 0/35 | NA | 0.99 | 0/35 | NA | 0.959 |
| **Hyperparathyroidism** | 0/335 | NA | 0.977 | 0/334 | NA | 0.987 | 13/319 | 2.03(1.18-3.49) | 0.011 |
| **Hyperpituitarism** | 1/195 | NA | 0.591 | 0/196 | NA | 0.984 | 1/194 | 0.31(0.04-2.19) | 0.24 |
| **Hypopituitarism** | 0/281 | NA | 0.979 | 0/281 | NA | 0.987 | 2/278 | 0.38(0.10-1.52) | 0.173 |
| **Cushing syndrome** | 0/83 | NA | 0.982 | 0/83 | NA | 0.985 | 2/79 | 1.54(0.38-6.14) | 0.544 |
| **Hyperaldosteronism** | 2/59 | NA | 0.003 | 0/61 | NA | 0.987 | 0/61 | NA | 0.945 |
| **Ovarian dysfunction** | 0/235 | NA | 0.974 | 0/235 | NA | 0.989 | 1/233 | 0.54(0.08-3.82) | 0.536 |
| **Testicular dysfunction** | 0/106 | NA | 0.98 | 0/106 | NA | 0.988 | 1/105 | 0.40(0.06-2.87) | 0.366 |

^$^Cox model was adjusted for sex, age, BMI, Townsend deprivation index, employment, education, ethnicity, physical activity, smoking status, drinking status, adherence to a healthy diet, social isolation, and C-reactive protein.

Abbreviations: HR: Hazard ratio, CI: Confidence interval, BMI: body mass index.

Table S9 HR (95% CI) of the number of endocrine comorbidities associated with total and individual gastrointestinal diseases^$^

| **Outcomes** | **Number of comorbidities** | **HR (95% CI)** | **P-value** | **HR (95% CI) for trend** | **P for trend** |
| --- | --- | --- | --- | --- | --- |
| Any gastrointestinal diseases | 1 | 1.50(1.46-1.55) | <.001 | 1.44(1.40-1.47) | <.001 |
|  | 2 | 1.65(1.47-1.85) | <.001 |  |  |
|  | ≥3 | 2.00(1.37-2.92) | <.001 |  |  |
| Non-neoplastic diseases | 1 | 1.53(1.48-1.58) | <.001 | 1.46(1.42-1.50) | <.001 |
|  | 2 | 1.69(1.51-1.89) | <.001 |  |  |
|  | ≥3 | 2.09(1.44-3.06) | <.001 |  |  |
| Gastric ulcer | 1 | 1.51(1.37-1.66) | <.001 | 1.45(1.33-1.57) | <.001 |
|  | 2 | 2.09(1.55-2.81) | <.001 |  |  |
|  | ≥3 | 0.46(0.07-3.28) | 0.441 |  |  |
| Duodenal ulcer | 1 | 1.51(1.33-1.72) | <.001 | 1.44(1.29-1.61) | <.001 |
|  | 2 | 1.64(1.02-2.65) | 0.042 |  |  |
|  | ≥3 | 2.09(0.52-8.36) | 0.298 |  |  |
| Gastritis and duodenitis | 1 | 1.60(1.54-1.67) | <.001 | 1.51(1.46-1.56) | <.001 |
|  | 2 | 1.79(1.55-2.07) | <.001 |  |  |
|  | ≥3 | 2.00(1.26-3.17) | 0.003 |  |  |
| Dyspepsia | 1 | 1.29(1.17-1.41) | <.001 | 1.23(1.14-1.33) | <.001 |
|  | 2 | 1.04(0.72-1.51) | 0.836 |  |  |
|  | ≥3 | 2.41(1.08-5.36) | 0.032 |  |  |
| Crohn disease | 1 | 1.32(1.06-1.65) | 0.013 | 1.34(1.12-1.60) | 0.002 |
|  | 2 | 2.22(1.22-4.03) | 0.009 |  |  |
|  | ≥3 | NA | 0.972 |  |  |
| Ulcerative colitis | 1 | 1.26(1.06-1.50) | 0.008 | 1.23(1.06-1.42) | 0.005 |
|  | 2 | 1.56(0.89-2.76) | 0.123 |  |  |
|  | ≥3 | NA | 0.971 |  |  |
| Irritable bowel syndrome | 1 | 1.44(1.33-1.57) | <.001 | 1.39(1.29-1.48) | <.001 |
|  | 2 | 1.66(1.26-2.19) | <.001 |  |  |
|  | ≥3 | 1.98(0.89-4.41) | 0.095 |  |  |
| Intestinal malabsorption | 1 | 1.69(1.43-1.99) | <.001 | 1.58(1.38-1.82) | <.001 |
|  | 2 | 2.32(1.37-3.92) | 0.002 |  |  |
|  | ≥3 | NA | 0.973 |  |  |
| Gastro-intestinal haemorrhage | 1 | 1.44(1.35-1.53) | <.001 | 1.41(1.34-1.48) | <.001 |
|  | 2 | 1.85(1.51-2.25) | <.001 |  |  |
|  | ≥3 | 2.34(1.33-4.13) | 0.003 |  |  |
| Neoplastic diseases | 1 | 0.97(0.88-1.07) | 0.534 | 0.96(0.88-1.05) | 0.376 |
|  | 2 | 0.92(0.62-1.37) | 0.693 |  |  |
|  | ≥3 | 0.42(0.06-3.01) | 0.391 |  |  |
| Gastric cancer | 1 | 1.19(0.95-1.48) | 0.124 | 1.10(0.90-1.34) | 0.359 |
|  | 2 | 0.67(0.21-2.07) | 0.484 |  |  |
|  | ≥3 | NA | 0.981 |  |  |
| Small intestine cancer | 1 | 1.20(0.81-1.79) | 0.364 | 1.10(0.77-1.57) | 0.598 |
|  | 2 | 0.67(0.09-4.77) | 0.688 |  |  |
|  | ≥3 | NA | 0.989 |  |  |
| Colorectal cancer | 1 | 0.90(0.80-1.01) | 0.082 | 0.92(0.83-1.01) | 0.086 |
|  | 2 | 0.96(0.62-1.47) | 0.851 |  |  |
|  | ≥3 | 0.53(0.07-3.76) | 0.525 |  |  |

^$^Cox model was adjusted for sex, age, BMI, Townsend deprivation index, employment, education, ethnicity, physical activity, smoking status, drinking status, adherence to a healthy diet, social isolation, and C-reactive protein.

Abbreviations: HR: Hazard ratio, CI: Confidence interval, BMI: body mass index.

Table S10 HR (95% CI) of gastrointestinal diseases associated with endocrine disease excluding missing data on covariates

| **exposure** | **outcome** | **case** | **control** | **HR (95% CI)** | **P-value** |
| --- | --- | --- | --- | --- | --- |
| Any gastrointestinal diseases | Any endocrine disease | 3987 | 19365 | 1.22(1.18-1.26) | <.001 |
| Any gastrointestinal diseases | Hypothalamic-pituitary-thyroid axis | 1884 | 22835 | 1.19(1.13-1.25) | <.001 |
| Any gastrointestinal diseases | Hypothalamic-pituitary-adrenal axis | 236 | 25453 | 1.56(1.35-1.79) | <.001 |
| Any gastrointestinal diseases | Hypothalamic-pituitary-gonadal axis | 152 | 25561 | 1.53(1.28-1.82) | <.001 |
| Any gastrointestinal diseases | Diseases of thyroid gland | 1825 | 22931 | 1.19(1.13-1.25) | <.001 |
| Any gastrointestinal diseases | Diseases of pancreatic gland | 2459 | 21947 | 1.22(1.17-1.28) | <.001 |
| Any gastrointestinal diseases | Diseases of parathyroid gland | 189 | 25541 | 1.56(1.33-1.82) | <.001 |
| Any gastrointestinal diseases | Diseases of hypothalamic-pituitary gland | 113 | 25624 | 1.52(1.24-1.86) | <.001 |
| Any gastrointestinal diseases | Diseases of adrenal gland | 141 | 25586 | 1.65(1.37-1.99) | <.001 |
| Any gastrointestinal diseases | Diseases of genital gland | 48 | 25708 | 1.62(1.19-2.22) | 0.002 |
| Any gastrointestinal diseases | Hypothyroidism | 1601 | 23331 | 1.19(1.13-1.26) | <.001 |
| Any gastrointestinal diseases | Hyperthyroidism | 292 | 25379 | 1.53(1.34-1.73) | <.001 |
| Any gastrointestinal diseases | Thyroiditis | 29 | 25732 | 1.28(0.87-1.90) | 0.213 |
| Any gastrointestinal diseases | Type 2 diabetes | 2384 | 22210 | 1.23(1.18-1.28) | <.001 |
| Any gastrointestinal diseases | Hypoparathyroidism | 14 | 25765 | 1.87(1.03-3.40) | 0.041 |
| Any gastrointestinal diseases | Hyperparathyroidism | 183 | 25551 | 1.57(1.34-1.85) | <.001 |
| Any gastrointestinal diseases | hyperpituitarism | 57 | 25704 | 1.27(0.96-1.69) | 0.093 |
| Any gastrointestinal diseases | hypopituitarism | 60 | 25691 | 1.84(1.38-2.44) | <.001 |
| Any gastrointestinal diseases | Cushing syndrome | 8 | 25758 | 1.02(0.48-2.14) | 0.967 |
| Any gastrointestinal diseases | Hyperaldosteronism | 9 | 25768 | 1.60(0.78-3.26) | 0.2 |
| Any gastrointestinal diseases | Ovarian dysfunction | 23 | 25744 | 1.76(1.12-2.75) | 0.014 |
| Any gastrointestinal diseases | Testicular dysfunction | 25 | 25747 | 1.54(1.00-2.37) | 0.049 |
| Non-neoplastic diseases | Any endocrine disease | 3846 | 18521 | 1.23(1.19-1.27) | <.001 |
| Gastric ulcer | Any endocrine disease | 221 | 916 | 1.14(1.00-1.31) | 0.046 |
| Duodenal ulcer | Any endocrine disease | 188 | 799 | 1.28(1.11-1.48) | <.001 |
| Gastritis and duodenitis | Any endocrine disease | 1582 | 6906 | 1.23(1.17-1.29) | <.001 |
| Dyspepsia | Any endocrine disease | 647 | 3325 | 1.16(1.07-1.26) | <.001 |
| Crohn disease | Any endocrine disease | 95 | 561 | 1.15(0.94-1.41) | 0.178 |
| Ulcerative colitis | Any endocrine disease | 180 | 1007 | 1.17(1.01-1.35) | 0.038 |
| Irritable bowel syndrome | Any endocrine disease | 251 | 1131 | 1.36(1.20-1.54) | <.001 |
| Intestinal malabsorption | Any endocrine disease | 74 | 426 | 1.22(0.97-1.53) | 0.09 |
| Gastro-intestinal haemorrhage | Any endocrine disease | 267 | 1202 | 1.21(1.07-1.36) | 0.002 |
| Neoplastic diseases | Any endocrine disease | 194 | 1191 | 0.93(0.81-1.07) | 0.337 |
| Gastric cancer | Any endocrine disease | 11 | 69 | 1.16(0.64-2.10) | 0.619 |
| Small intestine cancer | Any endocrine disease | 14 | 28 | 2.57(1.52-4.34) | <.001 |
| Colorectal cancer | Any endocrine disease | 175 | 1102 | 0.90(0.77-1.04) | 0.154 |

Cox model was adjusted for sex, age, BMI, Townsend deprivation index, employment, education, ethnicity, physical activity, smoking status, drinking status, adherence to a healthy diet, social isolation, and C-reactive protein.

Abbreviations: HR: Hazard ratio, CI: Confidence interval

Table S11 HR (95% CI) of gastrointestinal diseases associated with endocrine disease excluding the participants who developed within 1 years of follow-up.

| **exposure** | **outcome** | **case** | **control** | **HR (95% CI)** | **P-value** |
| --- | --- | --- | --- | --- | --- |
| Any gastrointestinal diseases | Any endocrine disease | 7637 | 36818 | 1.22(1.19-1.25) | <.001 |
| Any gastrointestinal diseases | Hypothalamic-pituitary-thyroid axis | 3519 | 43773 | 1.17(1.13-1.22) | <.001 |
| Any gastrointestinal diseases | Hypothalamic-pituitary-adrenal axis | 461 | 48847 | 1.53(1.38-1.69) | <.001 |
| Any gastrointestinal diseases | Hypothalamic-pituitary-gonadal axis | 286 | 49076 | 1.47(1.29-1.67) | <.001 |
| Any gastrointestinal diseases | Diseases of thyroid gland | 3396 | 43973 | 1.17(1.13-1.21) | <.001 |
| Any gastrointestinal diseases | Diseases of pancreatic gland | 4851 | 41761 | 1.24(1.20-1.28) | <.001 |
| Any gastrointestinal diseases | Diseases of parathyroid gland | 361 | 49041 | 1.45(1.30-1.63) | <.001 |
| Any gastrointestinal diseases | Diseases of hypothalamic-pituitary gland | 209 | 49208 | 1.43(1.23-1.66) | <.001 |
| Any gastrointestinal diseases | Diseases of adrenal gland | 287 | 49100 | 1.66(1.46-1.90) | <.001 |
| Any gastrointestinal diseases | Diseases of genital gland | 93 | 49357 | 1.66(1.33-2.09) | <.001 |
| Any gastrointestinal diseases | Hypothyroidism | 2960 | 44780 | 1.16(1.12-1.21) | <.001 |
| Any gastrointestinal diseases | Hyperthyroidism | 567 | 48705 | 1.50(1.37-1.64) | <.001 |
| Any gastrointestinal diseases | Thyroiditis | 55 | 49408 | 1.34(1.00-1.79) | 0.047 |
| Any gastrointestinal diseases | Type 2 diabetes | 4701 | 42298 | 1.24(1.20-1.28) | <.001 |
| Any gastrointestinal diseases | Hypoparathyroidism | 33 | 49467 | 1.84(1.23-2.73) | 0.003 |
| Any gastrointestinal diseases | Hyperparathyroidism | 345 | 49068 | 1.45(1.29-1.63) | <.001 |
| Any gastrointestinal diseases | hyperpituitarism | 126 | 49347 | 1.36(1.12-1.65) | 0.002 |
| Any gastrointestinal diseases | hypopituitarism | 91 | 49354 | 1.53(1.22-1.93) | <.001 |
| Any gastrointestinal diseases | Cushing syndrome | 24 | 49456 | 1.66(1.05-2.61) | 0.029 |
| Any gastrointestinal diseases | Hyperaldosteronism | 19 | 49482 | 2.04(1.22-3.40) | 0.006 |
| Any gastrointestinal diseases | Ovarian dysfunction | 40 | 49431 | 1.73(1.23-2.43) | 0.002 |
| Any gastrointestinal diseases | Testicular dysfunction | 52 | 49439 | 1.61(1.19-2.18) | 0.002 |
| Non-neoplastic diseases | Any endocrine disease | 7398 | 35311 | 1.23(1.20-1.27) | <.001 |
| Gastric ulcer | Any endocrine disease | 452 | 1841 | 1.16(1.05-1.27) | 0.002 |
| Duodenal ulcer | Any endocrine disease | 372 | 1637 | 1.22(1.10-1.35) | <.001 |
| Gastritis and duodenitis | Any endocrine disease | 2993 | 13334 | 1.20(1.16-1.25) | <.001 |
| Dyspepsia | Any endocrine disease | 1206 | 6167 | 1.16(1.10-1.23) | <.001 |
| Crohn disease | Any endocrine disease | 197 | 1026 | 1.23(1.07-1.41) | 0.004 |
| Ulcerative colitis | Any endocrine disease | 342 | 1915 | 1.18(1.06-1.31) | 0.003 |
| Irritable bowel syndrome | Any endocrine disease | 501 | 2255 | 1.33(1.22-1.45) | <.001 |
| Intestinal malabsorption | Any endocrine disease | 164 | 927 | 1.28(1.10-1.49) | 0.002 |
| Gastro-intestinal haemorrhage | Any endocrine disease | 545 | 2423 | 1.20(1.10-1.30) | <.001 |
| Neoplastic diseases | Any endocrine disease | 344 | 2144 | 0.92(0.83-1.03) | 0.15 |
| Gastric cancer | Any endocrine disease | 25 | 152 | 1.12(0.76-1.66) | 0.572 |
| Small intestine cancer | Any endocrine disease | 16 | 53 | 1.73(1.06-2.83) | 0.028 |
| Colorectal cancer | Any endocrine disease | 309 | 1960 | 0.90(0.80-1.00) | 0.058 |

Cox model was adjusted for sex, age, BMI, Townsend deprivation index, employment, education, ethnicity, physical activity, smoking status, drinking status, adherence to a healthy diet, social isolation, and C-reactive protein.

Abbreviations: HR: Hazard ratio, CI: Confidence interval

Table S12 HR (95% CI) of gastrointestinal diseases associated with endocrine disease excluding newly onset gastrointestinal disease

| **exposure** | **outcome** | **case** | **control** | **HR (95% CI)** | **P-value** |
| --- | --- | --- | --- | --- | --- |
| Any gastrointestinal diseases | Any endocrine disease | 8178 | 36277 | 1.51(1.47-1.55) | <.001 |
| Any gastrointestinal diseases | Hypothalamic-pituitary-thyroid axis | 3790 | 43502 | 1.45(1.40-1.50) | <.001 |
| Any gastrointestinal diseases | Hypothalamic-pituitary-adrenal axis | 477 | 48831 | 2.26(2.02-2.52) | <.001 |
| Any gastrointestinal diseases | Hypothalamic-pituitary-gonadal axis | 306 | 49056 | 2.18(1.90-2.49) | <.001 |
| Any gastrointestinal diseases | Diseases of thyroid gland | 3661 | 43708 | 1.44(1.38-1.49) | <.001 |
| Any gastrointestinal diseases | Diseases of pancreatic gland | 5163 | 41449 | 1.53(1.48-1.58) | <.001 |
| Any gastrointestinal diseases | Diseases of parathyroid gland | 378 | 49024 | 2.00(1.77-2.26) | <.001 |
| Any gastrointestinal diseases | Diseases of hypothalamic-pituitary gland | 220 | 49197 | 2.14(1.82-2.51) | <.001 |
| Any gastrointestinal diseases | Diseases of adrenal gland | 293 | 49094 | 2.45(2.12-2.82) | <.001 |
| Any gastrointestinal diseases | Diseases of genital gland | 103 | 49347 | 2.35(1.86-2.97) | <.001 |
| Any gastrointestinal diseases | Hypothyroidism | 3190 | 44550 | 1.42(1.37-1.48) | <.001 |
| Any gastrointestinal diseases | Hyperthyroidism | 606 | 48666 | 2.09(1.90-2.30) | <.001 |
| Any gastrointestinal diseases | Thyroiditis | 59 | 49404 | 1.77(1.31-2.37) | <.001 |
| Any gastrointestinal diseases | Type 2 diabetes | 5012 | 41987 | 1.54(1.49-1.59) | <.001 |
| Any gastrointestinal diseases | Hypoparathyroidism | 34 | 49466 | 3.05(1.95-4.78) | <.001 |
| Any gastrointestinal diseases | Hyperparathyroidism | 362 | 49051 | 2.00(1.76-2.26) | <.001 |
| Any gastrointestinal diseases | hyperpituitarism | 132 | 49341 | 2.12(1.73-2.61) | <.001 |
| Any gastrointestinal diseases | hypopituitarism | 97 | 49348 | 2.08(1.64-2.64) | <.001 |
| Any gastrointestinal diseases | Cushing syndrome | 24 | 49456 | 2.59(1.57-4.30) | <.001 |
| Any gastrointestinal diseases | Hyperaldosteronism | 20 | 49481 | 2.44(1.43-4.17) | 0.001 |
| Any gastrointestinal diseases | Ovarian dysfunction | 47 | 49424 | 2.46(1.75-3.46) | <.001 |
| Any gastrointestinal diseases | Testicular dysfunction | 55 | 49436 | 2.26(1.64-3.11) | <.001 |
| Non-neoplastic diseases | Any endocrine disease | 7920 | 34789 | 1.51(1.47-1.55) | <.001 |
| Gastric ulcer | Any endocrine disease | 487 | 1806 | 1.18(1.08-1.29) | <.001 |
| Duodenal ulcer | Any endocrine disease | 397 | 1612 | 1.23(1.11-1.36) | <.001 |
| Gastritis and duodenitis | Any endocrine disease | 3220 | 13107 | 1.30(1.25-1.35) | <.001 |
| Dyspepsia | Any endocrine disease | 1288 | 6085 | 1.18(1.11-1.24) | <.001 |
| Crohn disease | Any endocrine disease | 212 | 1011 | 1.24(1.09-1.42) | 0.001 |
| Ulcerative colitis | Any endocrine disease | 364 | 1893 | 1.18(1.07-1.31) | 0.002 |
| Irritable bowel syndrome | Any endocrine disease | 532 | 2224 | 1.35(1.24-1.47) | <.001 |
| Intestinal malabsorption | Any endocrine disease | 177 | 914 | 1.30(1.13-1.51) | <.001 |
| Gastro-intestinal haemorrhage | Any endocrine disease | 583 | 2385 | 1.24(1.14-1.34) | <.001 |
| Neoplastic diseases | Any endocrine disease | 367 | 2121 | 0.94(0.84-1.04) | 0.205 |
| Gastric cancer | Any endocrine disease | 26 | 151 | 1.09(0.74-1.61) | 0.648 |
| Small intestine cancer | Any endocrine disease | 17 | 52 | 1.71(1.06-2.75) | 0.027 |
| Colorectal cancer | Any endocrine disease | 330 | 1939 | 0.91(0.81-1.01) | 0.076 |

Cox model was adjusted for sex, age, BMI, Townsend deprivation index, employment, education, ethnicity, physical activity, smoking status, drinking status, adherence to a healthy diet, social isolation, and C-reactive protein.

Abbreviations: HR: Hazard ratio, CI: Confidence interval

Table S13 HR (95% CI) of endocrine disease associated with gastrointestinal diseases excluding missing data on covariates

| **exposure** | **outcome** | **case** | **control** | **HR (95% CI)** | **P-value** |
| --- | --- | --- | --- | --- | --- |
| Any endocrine disease | Any gastrointestinal diseases | 2913 | 5334 | 1.52(1.46-1.58) | <.001 |
| Any endocrine disease | Non-neoplastic diseases | 2857 | 5473 | 1.55(1.49-1.61) | <.001 |
| Any endocrine disease | Gastric ulcer | 298 | 10213 | 1.67(1.47-1.88) | <.001 |
| Any endocrine disease | Duodenal ulcer | 142 | 10419 | 1.43(1.20-1.71) | <.001 |
| Any endocrine disease | Gastritis and duodenitis | 1563 | 8196 | 1.67(1.58-1.76) | <.001 |
| Any endocrine disease | Dyspepsia | 306 | 10031 | 1.26(1.12-1.42) | <.001 |
| Any endocrine disease | Crohn disease | 53 | 10570 | 1.32(0.99-1.75) | 0.057 |
| Any endocrine disease | Ulcerative colitis | 93 | 10471 | 1.27(1.02-1.57) | 0.029 |
| Any endocrine disease | Irritable bowel syndrome | 369 | 10126 | 1.51(1.35-1.68) | <.001 |
| Any endocrine disease | Intestinal malabsorption | 102 | 10505 | 1.96(1.60-2.41) | <.001 |
| Any endocrine disease | Gastro-intestinal haemorrhage | 731 | 9767 | 1.48(1.37-1.60) | <.001 |
| Any endocrine disease | Neoplastic diseases | 260 | 10281 | 0.95(0.84-1.08) | 0.464 |
| Any endocrine disease | Gastric cancer | 53 | 10611 | 1.11(0.83-1.47) | 0.487 |
| Any endocrine disease | Small intestine cancer | 18 | 10649 | 1.21(0.74-1.97) | 0.445 |
| Any endocrine disease | Colorectal cancer | 197 | 10366 | 0.90(0.77-1.04) | 0.135 |
| Hypothalamic-pituitary-thyroid axis | Any gastrointestinal diseases | 1269 | 2565 | 1.46(1.38-1.55) | <.001 |
| Hypothalamic-pituitary-adrenal axis | Any gastrointestinal diseases | 110 | 210 | 1.67(1.38-2.01) | <.001 |
| Hypothalamic-pituitary-gonadal axis | Any gastrointestinal diseases | 80 | 243 | 1.27(1.02-1.58) | 0.035 |
| Diseases of thyroid gland | Any gastrointestinal diseases | 1227 | 2456 | 1.47(1.39-1.55) | <.001 |
| Diseases of pancreatic gland | Any gastrointestinal diseases | 1679 | 2686 | 1.55(1.48-1.63) | <.001 |
| Diseases of parathyroid gland | Any gastrointestinal diseases | 55 | 85 | 1.84(1.41-2.40) | <.001 |
| Diseases of hypothalamic-pituitary gland | Any gastrointestinal diseases | 51 | 125 | 1.36(1.03-1.79) | 0.027 |
| Diseases of adrenal gland | Any gastrointestinal diseases | 65 | 90 | 2.13(1.67-2.72) | <.001 |
| Diseases of genital gland | Any gastrointestinal diseases | 31 | 124 | 1.13(0.79-1.61) | 0.495 |
| Hypothyroidism | Any gastrointestinal diseases | 945 | 1776 | 1.50(1.41-1.60) | <.001 |
| Hyperthyroidism | Any gastrointestinal diseases | 147 | 322 | 1.42(1.21-1.67) | <.001 |
| Thyroiditis | Any gastrointestinal diseases | 29 | 55 | 1.65(1.15-2.38) | 0.007 |
| Type 2 diabetes | Any gastrointestinal diseases | 1445 | 2148 | 1.57(1.49-1.66) | <.001 |
| Hypoparathyroidism | Any gastrointestinal diseases | 5 | 3 | 3.80(1.58-9.13) | 0.003 |
| Hyperparathyroidism | Any gastrointestinal diseases | 50 | 82 | 1.75(1.33-2.31) | <.001 |
| hyperpituitarism | Any gastrointestinal diseases | 23 | 53 | 1.46(0.97-2.20) | 0.068 |
| hypopituitarism | Any gastrointestinal diseases | 32 | 79 | 1.33(0.94-1.88) | 0.105 |
| Cushing syndrome | Any gastrointestinal diseases | 13 | 18 | 1.94(1.13-3.35) | 0.017 |
| Hyperaldosteronism | Any gastrointestinal diseases | 12 | 12 | 2.98(1.69-5.24) | <.001 |
| Ovarian dysfunction | Any gastrointestinal diseases | 22 | 85 | 1.31(0.86-1.99) | 0.203 |
| Testicular dysfunction | Any gastrointestinal diseases | 9 | 39 | 0.84(0.44-1.62) | 0.613 |

Cox model was adjusted for sex, age, BMI, Townsend deprivation index, employment, education, ethnicity, physical activity, smoking status, drinking status, adherence to a healthy diet, social isolation, and C-reactive protein.

Abbreviations: HR: Hazard ratio, CI: Confidence interval

Table S14 HR (95% CI) of endocrine disease associated with gastrointestinal diseases excluding the participants who developed within 1 years of follow-up.

| **exposure** | **outcome** | **case** | **control** | **HR (95% CI)** | **P-value** |
| --- | --- | --- | --- | --- | --- |
| Any endocrine disease | Any gastrointestinal diseases | 5123 | 10349 | 1.47(1.42-1.51) | <.001 |
| Any endocrine disease | Non-neoplastic diseases | 5044 | 10595 | 1.49(1.45-1.54) | <.001 |
| Any endocrine disease | Gastric ulcer | 532 | 19634 | 1.52(1.38-1.66) | <.001 |
| Any endocrine disease | Duodenal ulcer | 276 | 20003 | 1.43(1.26-1.62) | <.001 |
| Any endocrine disease | Gastritis and duodenitis | 2799 | 15800 | 1.56(1.50-1.63) | <.001 |
| Any endocrine disease | Dyspepsia | 547 | 19323 | 1.26(1.15-1.37) | <.001 |
| Any endocrine disease | Crohn disease | 105 | 20298 | 1.37(1.11-1.67) | 0.003 |
| Any endocrine disease | Ulcerative colitis | 171 | 20137 | 1.27(1.08-1.48) | 0.004 |
| Any endocrine disease | Irritable bowel syndrome | 697 | 19445 | 1.38(1.28-1.50) | <.001 |
| Any endocrine disease | Intestinal malabsorption | 190 | 20177 | 1.77(1.52-2.06) | <.001 |
| Any endocrine disease | Gastro-intestinal haemorrhage | 1393 | 18694 | 1.42(1.34-1.50) | <.001 |
| Any endocrine disease | Neoplastic diseases | 475 | 19776 | 0.96(0.87-1.05) | 0.391 |
| Any endocrine disease | Gastric cancer | 97 | 20404 | 1.13(0.92-1.40) | 0.252 |
| Any endocrine disease | Small intestine cancer | 30 | 20482 | 1.15(0.78-1.67) | 0.484 |
| Any endocrine disease | Colorectal cancer | 363 | 19928 | 0.91(0.82-1.01) | 0.079 |
| Hypothalamic-pituitary-thyroid axis | Any gastrointestinal diseases | 2156 | 4940 | 1.37(1.32-1.43) | <.001 |
| Hypothalamic-pituitary-adrenal axis | Any gastrointestinal diseases | 173 | 418 | 1.39(1.20-1.61) | <.001 |
| Hypothalamic-pituitary-gonadal axis | Any gastrointestinal diseases | 147 | 465 | 1.21(1.03-1.42) | 0.022 |
| Diseases of thyroid gland | Any gastrointestinal diseases | 2077 | 4713 | 1.38(1.32-1.44) | <.001 |
| Diseases of pancreatic gland | Any gastrointestinal diseases | 3016 | 5298 | 1.52(1.47-1.58) | <.001 |
| Diseases of parathyroid gland | Any gastrointestinal diseases | 89 | 167 | 1.69(1.37-2.08) | <.001 |
| Diseases of hypothalamic-pituitary gland | Any gastrointestinal diseases | 92 | 258 | 1.22(1.00-1.50) | 0.052 |
| Diseases of adrenal gland | Any gastrointestinal diseases | 91 | 178 | 1.66(1.35-2.04) | <.001 |
| Diseases of genital gland | Any gastrointestinal diseases | 59 | 220 | 1.18(0.92-1.53) | 0.194 |
| Hypothyroidism | Any gastrointestinal diseases | 1607 | 3399 | 1.43(1.36-1.50) | <.001 |
| Hyperthyroidism | Any gastrointestinal diseases | 240 | 621 | 1.28(1.13-1.46) | <.001 |
| Thyroiditis | Any gastrointestinal diseases | 48 | 109 | 1.44(1.08-1.91) | 0.012 |
| Type 2 diabetes | Any gastrointestinal diseases | 2610 | 4304 | 1.54(1.48-1.61) | <.001 |
| Hypoparathyroidism | Any gastrointestinal diseases | 9 | 14 | 2.23(1.16-4.29) | 0.016 |
| Hyperparathyroidism | Any gastrointestinal diseases | 81 | 155 | 1.65(1.32-2.05) | <.001 |
| hyperpituitarism | Any gastrointestinal diseases | 36 | 121 | 1.13(0.82-1.57) | 0.461 |
| hypopituitarism | Any gastrointestinal diseases | 60 | 154 | 1.25(0.97-1.61) | 0.085 |
| Cushing syndrome | Any gastrointestinal diseases | 14 | 37 | 1.10(0.65-1.86) | 0.724 |
| Hyperaldosteronism | Any gastrointestinal diseases | 22 | 28 | 2.26(1.49-3.44) | <.001 |
| Ovarian dysfunction | Any gastrointestinal diseases | 41 | 153 | 1.34(0.99-1.82) | 0.06 |
| Testicular dysfunction | Any gastrointestinal diseases | 18 | 67 | 0.93(0.59-1.48) | 0.773 |

Cox model was adjusted for sex, age, BMI, Townsend deprivation index, employment, education, ethnicity, physical activity, smoking status, drinking status, adherence to a healthy diet, social isolation, and C-reactive protein.

Abbreviations: HR: Hazard ratio, CI: Confidence interval

Table S15 HR (95% CI) of endocrine disease associated with gastrointestinal diseases excluding newly onset endocrine disease

| **exposure** | **outcome** | **case** | **control** | **HR (95% CI)** | **P-value** |
| --- | --- | --- | --- | --- | --- |
| Any endocrine disease | Any gastrointestinal diseases | 5609 | 9863 | 1.71(1.66-1.75) | <.001 |
| Any endocrine disease | Non-neoplastic diseases | 5522 | 10117 | 1.74(1.69-1.79) | <.001 |
| Any endocrine disease | Gastric ulcer | 582 | 19584 | 1.80(1.64-1.97) | <.001 |
| Any endocrine disease | Duodenal ulcer | 303 | 19976 | 1.69(1.49-1.91) | <.001 |
| Any endocrine disease | Gastritis and duodenitis | 3035 | 15564 | 1.82(1.75-1.90) | <.001 |
| Any endocrine disease | Dyspepsia | 599 | 19271 | 1.33(1.22-1.45) | <.001 |
| Any endocrine disease | Crohn disease | 108 | 20295 | 1.51(1.23-1.85) | <.001 |
| Any endocrine disease | Ulcerative colitis | 182 | 20126 | 1.44(1.23-1.68) | <.001 |
| Any endocrine disease | Irritable bowel syndrome | 735 | 19407 | 1.64(1.52-1.78) | <.001 |
| Any endocrine disease | Intestinal malabsorption | 204 | 20163 | 2.09(1.80-2.42) | <.001 |
| Any endocrine disease | Gastro-intestinal haemorrhage | 1460 | 18627 | 1.65(1.56-1.75) | <.001 |
| Any endocrine disease | Neoplastic diseases | 510 | 19741 | 1.08(0.99-1.19) | 0.087 |
| Any endocrine disease | Gastric cancer | 101 | 20400 | 1.27(1.02-1.57) | 0.031 |
| Any endocrine disease | Small intestine cancer | 30 | 20482 | 1.35(0.91-2.00) | 0.132 |
| Any endocrine disease | Colorectal cancer | 394 | 19897 | 1.03(0.93-1.14) | 0.603 |
| Hypothalamic-pituitary-thyroid axis | Any gastrointestinal diseases | 2365 | 4731 | 1.47(1.41-1.54) | <.001 |
| Hypothalamic-pituitary-adrenal axis | Any gastrointestinal diseases | 190 | 401 | 1.43(1.24-1.65) | <.001 |
| Hypothalamic-pituitary-gonadal axis | Any gastrointestinal diseases | 161 | 451 | 1.24(1.06-1.44) | 0.007 |
| Diseases of thyroid gland | Any gastrointestinal diseases | 2279 | 4511 | 1.48(1.42-1.54) | <.001 |
| Diseases of pancreatic gland | Any gastrointestinal diseases | 3299 | 5015 | 1.71(1.65-1.77) | <.001 |
| Diseases of parathyroid gland | Any gastrointestinal diseases | 103 | 153 | 1.82(1.50-2.20) | <.001 |
| Diseases of hypothalamic-pituitary gland | Any gastrointestinal diseases | 101 | 249 | 1.25(1.03-1.52) | 0.023 |
| Diseases of adrenal gland | Any gastrointestinal diseases | 101 | 168 | 1.72(1.41-2.09) | <.001 |
| Diseases of genital gland | Any gastrointestinal diseases | 65 | 214 | 1.22(0.95-1.55) | 0.117 |
| Hypothyroidism | Any gastrointestinal diseases | 1772 | 3234 | 1.53(1.45-1.60) | <.001 |
| Hyperthyroidism | Any gastrointestinal diseases | 260 | 601 | 1.30(1.15-1.47) | <.001 |
| Thyroiditis | Any gastrointestinal diseases | 49 | 108 | 1.37(1.04-1.81) | 0.028 |
| Type 2 diabetes | Any gastrointestinal diseases | 2851 | 4063 | 1.73(1.67-1.80) | <.001 |
| Hypoparathyroidism | Any gastrointestinal diseases | 10 | 13 | 2.26(1.22-4.20) | 0.01 |
| Hyperparathyroidism | Any gastrointestinal diseases | 95 | 141 | 1.80(1.47-2.20) | <.001 |
| hyperpituitarism | Any gastrointestinal diseases | 43 | 114 | 1.26(0.93-1.70) | 0.131 |
| hypopituitarism | Any gastrointestinal diseases | 63 | 151 | 1.22(0.95-1.57) | 0.111 |
| Cushing syndrome | Any gastrointestinal diseases | 16 | 35 | 1.17(0.72-1.91) | 0.53 |
| Hyperaldosteronism | Any gastrointestinal diseases | 24 | 26 | 2.29(1.53-3.42) | <.001 |
| Ovarian dysfunction | Any gastrointestinal diseases | 45 | 149 | 1.37(1.02-1.83) | 0.035 |
| Testicular dysfunction | Any gastrointestinal diseases | 20 | 65 | 0.97(0.63-1.50) | 0.888 |

Cox model was adjusted for sex, age, BMI, Townsend deprivation index, employment, education, ethnicity, physical activity, smoking status, drinking status, adherence to a healthy diet, social isolation, and C-reactive protein.

Abbreviations: HR: Hazard ratio, CI: Confidence interval

Table S16 HR (95% CI) of any gastrointestinal diseases associated with total and individual endocrine disease according to baseline type 2 diabetes stratified by age, sex, body mass index, smoking status, and drinking status^$^

| **Outcomes** | **Male** |  | **Female** |  | **P-interaction** | **Age 60≤ years** | | **Age >60 years** | | **P-interaction** |
| --- | --- | --- | --- | --- | --- | --- | --- | --- | --- | --- |
|  | **HR (95% CI)** | **P value** | **HR (95% CI)** | **P value** |  | **HR (95% CI)** | **P value** | **HR (95% CI)** | **P value** |  |
| **Any endocrine disease** | 1.19(1.15-1.24) | <.001 | 1.24(1.21-1.28) | <.001 | <.001 | 1.31(1.26-1.36) | <.001 | 1.16(1.12-1.19) | <.001 | <.001 |
| **Hypothalamic-pituitary-thyroid axis** | 1.22(1.14-1.31) | <.001 | 1.17(1.12-1.22) | <.001 | <.001 | 1.26(1.20-1.33) | <.001 | 1.12(1.07-1.18) | <.001 | <.001 |
| **Hypothalamic-pituitary-adrenal axis** | 1.55(1.34-1.79) | <.001 | 1.50(1.31-1.73) | <.001 | 0.926 | 1.69(1.45-1.96) | <.001 | 1.42(1.24-1.62) | <.001 | <.001 |
| **Hypothalamic-pituitary-gonadal axis** | 1.47(1.23-1.74) | <.001 | 1.56(1.31-1.88) | <.001 | 0.01 | 1.63(1.37-1.95) | <.001 | 1.41(1.19-1.68) | <.001 | 0.357 |
| **Diseases of thyroid gland** | 1.21(1.13-1.31) | <.001 | 1.17(1.12-1.22) | <.001 | <.001 | 1.25(1.19-1.32) | <.001 | 1.12(1.07-1.17) | <.001 | <.001 |
| **Diseases of pancreatic gland** | 1.17(1.12-1.22) | <.001 | 1.30(1.24-1.35) | <.001 | <.001 | 1.32(1.26-1.38) | <.001 | 1.16(1.11-1.21) | <.001 | <.001 |
| **Diseases of parathyroid gland** | 1.69(1.37-2.10) | <.001 | 1.41(1.23-1.61) | <.001 | <.001 | 1.78(1.50-2.11) | <.001 | 1.29(1.11-1.50) | 0.001 | <.001 |
| **Diseases of hypothalamic-pituitary gland** | 1.43(1.17-1.75) | <.001 | 1.47(1.19-1.83) | <.001 | <.001 | 1.56(1.24-1.96) | <.001 | 1.39(1.14-1.68) | <.001 | <.001 |
| **Diseases of adrenal gland** | 1.70(1.40-2.07) | <.001 | 1.59(1.33-1.89) | <.001 | <.001 | 1.82(1.51-2.18) | <.001 | 1.49(1.24-1.79) | <.001 | <.001 |
| **Diseases of genital gland** | 1.65(1.23-2.22) | <.001 | 1.89(1.37-2.59) | <.001 | 0.955 | 1.77(1.36-2.30) | <.001 | 1.71(1.16-2.51) | 0.006 | <.001 |
| **Hypothyroidism** | 1.23(1.13-1.33) | <.001 | 1.15(1.10-1.20) | <.001 | <.001 | 1.25(1.18-1.32) | <.001 | 1.10(1.05-1.16) | <.001 | <.001 |
| **Hyperthyroidism** | 1.34(1.11-1.63) | 0.003 | 1.60(1.45-1.77) | <.001 | <.001 | 1.67(1.47-1.91) | <.001 | 1.44(1.27-1.62) | <.001 | <.001 |
| **Thyroiditis** | 1.57(0.76-3.22) | 0.222 | 1.34(0.99-1.81) | 0.059 | <.001 | 1.37(0.95-1.98) | 0.096 | 1.38(0.90-2.12) | 0.139 | 0.197 |
| **Type 2 diabetes** | 1.17(1.12-1.22) | <.001 | 1.30(1.25-1.36) | <.001 | <.001 | 1.33(1.27-1.39) | <.001 | 1.16(1.11-1.21) | <.001 | <.001 |
| **Hypoparathyroidism** | 1.81(0.94-3.51) | 0.077 | 1.89(1.16-3.08) | 0.01 | 0.077 | 2.19(1.27-3.79) | 0.005 | 1.60(0.92-2.81) | 0.099 | 0.024 |
| **Hyperparathyroidism** | 1.74(1.40-2.17) | <.001 | 1.40(1.22-1.60) | <.001 | <.001 | 1.80(1.51-2.13) | <.001 | 1.28(1.10-1.50) | 0.002 | <.001 |
| **Hyperpituitarism** | 1.34(1.02-1.75) | 0.037 | 1.46(1.12-1.90) | 0.005 | 0.055 | 1.78(1.31-2.42) | <.001 | 1.23(0.97-1.57) | 0.087 | <.001 |
| **Hypopituitarism** | 1.56(1.18-2.07) | 0.002 | 1.46(1.01-2.10) | 0.041 | <.001 | 1.34(0.97-1.85) | 0.076 | 1.73(1.28-2.36) | <.001 | 0.77 |
| **Cushing syndrome** | 1.76(0.71-4.33) | 0.221 | 1.54(0.91-2.60) | 0.106 | <.001 | 2.16(1.19-3.94) | 0.012 | 1.12(0.56-2.24) | 0.743 | 0.034 |
| **Hyperaldosteronism** | 1.74(0.90-3.37) | 0.1 | 2.51(1.17-5.42) | 0.019 | 0.003 | 1.83(0.95-3.54) | 0.072 | 2.36(1.10-5.07) | 0.028 | 0.113 |
| **Ovarian dysfunction** | NA | 0.872 | 1.86(1.35-2.56) | <.001 | <.001 | 1.84(1.31-2.59) | <.001 | 1.71(0.68-4.29) | 0.253 | <.001 |
| **Testicular dysfunction** | 1.68(1.25-2.27) | <.001 | NA | 1 | <.001 | 1.70(1.13-2.56) | 0.012 | 1.67(1.09-2.56) | 0.02 | 0.658 |

| **Outcome** | **Ever smoker** | | **Non smoker** | | **P-interaction** | **Current drinker** | | **Non-current drinker** | | **P-interaction** |
| --- | --- | --- | --- | --- | --- | --- | --- | --- | --- | --- |
|  | **HR (95% CI)** | **P value** | **HR (95% CI)** | **P value** |  | **HR (95% CI)** | **P value** | **HR (95% CI)** | **P value** |  |
| **Any endocrine disease** | 1.19(1.15-1.23) | <.001 | 1.26(1.22-1.31) | <.001 | <.001 | 1.23(1.20-1.26) | <.001 | 1.14(1.05-1.24) | 0.002 | <.001 |
| **Hypothalamic-pituitary-thyroid axis** | 1.13(1.08-1.19) | <.001 | 1.24(1.18-1.30) | <.001 | <.001 | 1.19(1.15-1.24) | <.001 | 1.06(0.94-1.20) | 0.321 | 0.977 |
| **Hypothalamic-pituitary-adrenal axis** | 1.45(1.27-1.66) | <.001 | 1.64(1.41-1.91) | <.001 | 0.127 | 1.49(1.34-1.66) | <.001 | 2.00(1.43-2.80) | <.001 | 0.681 |
| **Hypothalamic-pituitary-gonadal axis** | 1.45(1.23-1.72) | <.001 | 1.58(1.31-1.89) | <.001 | 0.104 | 1.45(1.27-1.65) | <.001 | 2.31(1.54-3.45) | <.001 | 0.795 |
| **Diseases of thyroid gland** | 1.12(1.07-1.18) | <.001 | 1.24(1.18-1.30) | <.001 | <.001 | 1.19(1.15-1.24) | <.001 | 1.04(0.92-1.18) | 0.525 | 0.998 |
| **Diseases of pancreatic gland** | 1.22(1.18-1.27) | <.001 | 1.24(1.18-1.29) | <.001 | <.001 | 1.24(1.20-1.28) | <.001 | 1.17(1.06-1.29) | 0.002 | <.001 |
| **Diseases of parathyroid gland** | 1.52(1.29-1.78) | <.001 | 1.46(1.24-1.71) | <.001 | 0.295 | 1.51(1.34-1.69) | <.001 | 1.23(0.84-1.82) | 0.286 | 0.703 |
| **Diseases of hypothalamic-pituitary gland** | 1.38(1.13-1.69) | 0.002 | 1.54(1.24-1.91) | <.001 | 0.501 | 1.42(1.22-1.66) | <.001 | 1.77(1.09-2.87) | 0.021 | 0.449 |
| **Diseases of adrenal gland** | 1.56(1.31-1.85) | <.001 | 1.75(1.44-2.14) | <.001 | 0.41 | 1.61(1.41-1.84) | <.001 | 1.99(1.27-3.12) | 0.003 | 0.277 |
| **Diseases of genital gland** | 1.73(1.29-2.34) | <.001 | 1.76(1.28-2.41) | <.001 | 0.163 | 1.60(1.27-2.02) | <.001 | 4.36(2.20-8.66) | <.001 | 0.35 |
| **Hypothyroidism** | 1.12(1.06-1.18) | <.001 | 1.22(1.16-1.29) | <.001 | <.001 | 1.18(1.13-1.23) | <.001 | 1.04(0.91-1.19) | 0.586 | 0.719 |
| **Hyperthyroidism** | 1.50(1.33-1.69) | <.001 | 1.59(1.39-1.81) | <.001 | <.001 | 1.56(1.43-1.72) | <.001 | 1.25(0.90-1.74) | 0.189 | 0.901 |
| **Thyroiditis** | 1.41(0.94-2.12) | 0.096 | 1.31(0.89-1.93) | 0.165 | 0.409 | 1.37(1.02-1.83) | 0.037 | 1.32(0.54-3.25) | 0.542 | 0.993 |
| **Type 2 diabetes** | 1.22(1.18-1.27) | <.001 | 1.25(1.19-1.31) | <.001 | <.001 | 1.25(1.21-1.29) | <.001 | 1.16(1.05-1.28) | 0.005 | <.001 |
| **Hypoparathyroidism** | 2.16(1.26-3.70) | 0.005 | 1.61(0.91-2.86) | 0.102 | 0.96 | 1.81(1.20-2.74) | 0.005 | 2.24(0.65-7.69) | 0.2 | 0.75 |
| **Hyperparathyroidism** | 1.50(1.27-1.77) | <.001 | 1.48(1.26-1.74) | <.001 | 0.31 | 1.51(1.34-1.70) | <.001 | 1.21(0.81-1.80) | 0.354 | 0.731 |
| **Hyperpituitarism** | 1.21(0.93-1.56) | 0.157 | 1.68(1.27-2.21) | <.001 | 0.036 | 1.38(1.13-1.68) | 0.001 | 1.61(0.82-3.18) | 0.165 | 0.974 |
| **Hypopituitarism** | 1.71(1.26-2.33) | <.001 | 1.36(0.99-1.88) | 0.061 | 0.178 | 1.50(1.18-1.89) | <.001 | 1.80(0.90-3.58) | 0.096 | 0.521 |
| **Cushing syndrome** | 1.43(0.77-2.65) | 0.259 | 1.75(0.90-3.40) | 0.098 | 0.533 | 1.48(0.92-2.36) | 0.103 | NA | <.001 | 0.02 |
| **Hyperaldosteronism** | 1.58(0.72-3.48) | 0.252 | 2.36(1.24-4.48) | 0.009 | 0.196 | 1.73(1.00-2.99) | 0.051 | NA | 0.009 | 0.903 |
| **Ovarian dysfunction** | 1.90(1.17-3.10) | 0.01 | 1.80(1.18-2.75) | 0.007 | 0.773 | 1.69(1.19-2.39) | 0.003 | 3.92(1.64-9.38) | 0.002 | 0.325 |
| **Testicular dysfunction** | 1.63(1.11-2.39) | 0.012 | 1.75(1.09-2.81) | 0.02 | 0.454 | 1.54(1.13-2.11) | 0.006 | NA | 0.002 | 0.431 |

| **Outcomes** | **Obesity** |  | **Non-obesity** | | **P-interaction** |
| --- | --- | --- | --- | --- | --- |
|  | **HR (95% CI)** | **P value** | **HR (95% CI)** | **P value** |  |
| **Any endocrine disease** | 1.16(1.13-1.20) | <.001 | 1.31(1.26-1.35) | <.001 | <.001 |
| **Hypothalamic-pituitary-thyroid axis** | 1.13(1.08-1.19) | <.001 | 1.24(1.18-1.30) | <.001 | <.001 |
| **Hypothalamic-pituitary-adrenal axis** | 1.48(1.28-1.72) | <.001 | 1.56(1.36-1.80) | <.001 | 0.04 |
| **Hypothalamic-pituitary-gonadal axis** | 1.47(1.23-1.76) | <.001 | 1.55(1.30-1.84) | <.001 | <.001 |
| **Diseases of thyroid gland** | 1.13(1.07-1.18) | <.001 | 1.23(1.18-1.30) | <.001 | <.001 |
| **Diseases of pancreatic gland** | 1.16(1.11-1.20) | <.001 | 1.36(1.29-1.44) | <.001 | <.001 |
| **Diseases of parathyroid gland** | 1.42(1.21-1.66) | <.001 | 1.55(1.32-1.81) | <.001 | <.001 |
| **Diseases of hypothalamic-pituitary gland** | 1.45(1.16-1.81) | 0.001 | 1.45(1.19-1.76) | <.001 | 0.184 |
| **Diseases of adrenal gland** | 1.49(1.24-1.79) | <.001 | 1.80(1.50-2.16) | <.001 | <.001 |
| **Diseases of genital gland** | 1.64(1.25-2.16) | <.001 | 1.94(1.36-2.77) | <.001 | <.001 |
| **Hypothyroidism** | 1.10(1.04-1.16) | <.001 | 1.24(1.17-1.30) | <.001 | <.001 |
| **Hyperthyroidism** | 1.53(1.35-1.74) | <.001 | 1.55(1.37-1.75) | <.001 | 0.001 |
| **Thyroiditis** | 1.26(0.81-1.96) | 0.307 | 1.46(1.02-2.10) | 0.038 | 0.807 |
| **Type 2 diabetes** | 1.16(1.12-1.20) | <.001 | 1.37(1.29-1.45) | <.001 | <.001 |
| **Hypoparathyroidism** | 1.38(0.76-2.50) | 0.297 | 2.40(1.43-4.02) | <.001 | 0.622 |
| **Hyperparathyroidism** | 1.42(1.21-1.68) | <.001 | 1.54(1.31-1.82) | <.001 | <.001 |
| **Hyperpituitarism** | 1.28(0.92-1.77) | 0.146 | 1.46(1.16-1.84) | 0.001 | <.001 |
| **Hypopituitarism** | 1.58(1.18-2.11) | 0.002 | 1.44(1.02-2.05) | 0.039 | <.001 |
| **Cushing syndrome** | 1.48(0.84-2.58) | 0.174 | 1.72(0.80-3.72) | 0.167 | <.001 |
| **Hyperaldosteronism** | 1.23(0.60-2.51) | 0.578 | 3.88(1.91-7.89) | <.001 | <.001 |
| **Ovarian dysfunction** | 1.69(1.12-2.55) | 0.012 | 2.05(1.23-3.40) | 0.006 | <.001 |
| **Testicular dysfunction** | 1.60(1.10-2.32) | 0.014 | 1.85(1.13-3.02) | 0.015 | <.001 |

^$^Cox model was adjusted for sex, age, BMI, Townsend deprivation index, employment, education, ethnicity, physical activity, smoking status, drinking status, adherence to a healthy diet, social isolation, and C-reactive protein except for stratified factors.

Abbreviations: HR: Hazard ratio, CI: Confidence interval, BMI: body mass index.

Table S17 HR (95% CI) of any gastrointestinal diseases associated with total and individual endocrine disease according to medication use^$^

| **Outcomes** | **medication use** | **Case No./control No.** | | | **HR (95% CI)** | **P value** |
| --- | --- | --- | --- | --- | --- | --- |
|  |  | **Total** | **With**  **gastrointestinal diseases** | **Without**  **gastrointestinal diseases** |  |  |
| **Any endocrine disease** | Yes | 14651/58466 | 4278/15448 | 10373/43018 | 1.05(1.02-1.09) | 0.004 |
| **Hypothalamic-pituitary-thyroid axis** | Yes | 6581/70765 | 1868/19606 | 4713/51159 | 1.00(0.95-1.05) | 0.972 |
| **Hypothalamic-pituitary-adrenal axis** | Yes | 893/79102 | 276/22266 | 617/56836 | 1.06(0.92-1.22) | 0.418 |
| **Hypothalamic-pituitary-gonadal axis** | Yes | 580/79550 | 182/22423 | 398/57127 | 1.13(0.95-1.35) | 0.178 |
| **Diseases of thyroid gland** | Yes | 6354/71192 | 1809/19726 | 4545/51466 | 1.00(0.95-1.06) | 0.891 |
| **Diseases of pancreatic gland** | Yes | 9533/66625 | 2928/17978 | 6605/48647 | 1.08(1.03-1.13) | <.001 |
| **Diseases of parathyroid gland** | Yes | 634/79674 | 232/22407 | 402/57267 | 1.37(1.17-1.62) | <.001 |
| **Diseases of hypothalamic-pituitary gland** | Yes | 425/79789 | 124/22511 | 301/57278 | 0.99(0.81-1.23) | 0.958 |
| **Diseases of adrenal gland** | Yes | 556/79643 | 182/22421 | 374/57222 | 1.14(0.96-1.37) | 0.145 |
| **Diseases of genital gland** | Yes | 188/80163 | 67/22607 | 121/57556 | 1.45(1.07-1.96) | 0.017 |
| **Hypothyroidism** | Yes | 5608/72465 | 1563/20156 | 4045/52309 | 0.97(0.92-1.03) | 0.331 |
| **Hyperthyroidism** | Yes | 921/79215 | 338/22251 | 583/56964 | 1.38(1.20-1.58) | <.001 |
| **Thyroiditis** | Yes | 102/80288 | 27/22658 | 75/57630 | 0.98(0.63-1.52) | 0.918 |
| **Type 2 diabetes** | Yes | 9281/67378 | 2851/18255 | 6430/49123 | 1.07(1.03-1.12) | 0.002 |
| **Hypoparathyroidism** | Yes | 62/80371 | 25/22674 | 37/57697 | 1.44(0.86-2.41) | 0.162 |
| **Hyperparathyroidism** | Yes | 599/79726 | 220/22430 | 379/57296 | 1.39(1.18-1.65) | <.001 |
| **Hyperpituitarism** | Yes | 230/80132 | 73/22607 | 157/57525 | 1.09(0.82-1.44) | 0.567 |
| **Hypopituitarism** | Yes | 222/80057 | 58/22597 | 164/57460 | 0.89(0.66-1.20) | 0.445 |
| **Cushing syndrome** | Yes | 50/80344 | 16/22665 | 34/57679 | 1.14(0.62-2.07) | 0.679 |
| **Hyperaldosteronism** | Yes | 27/80408 | 10/22695 | 17/57713 | 1.47(0.66-3.24) | 0.343 |
| **Ovarian dysfunction** | Yes | 86/80307 | 33/22658 | 53/57649 | 1.76(1.13-2.74) | 0.012 |
| **Testicular dysfunction** | Yes | 99/80311 | 33/22661 | 66/57650 | 1.20(0.79-1.83) | 0.394 |

| **Outcomes** | **medication use** | **Case No./control No.** | | | **HR (95% CI)** | **P value** |
| --- | --- | --- | --- | --- | --- | --- |
|  |  | **Total** | **With**  **gastrointestinal diseases** | **Without**  **gastrointestinal diseases** |  |  |
| **Any endocrine disease** | No | 46717/362007 | 3900/20829 | 42817/341178 | 1.22(1.18-1.26) | <.001 |
| **Hypothalamic-pituitary-thyroid axis** | No | 22380/393328 | 1922/23896 | 20458/369432 | 1.23(1.17-1.29) | <.001 |
| **Hypothalamic-pituitary-adrenal axis** | No | 1877/419703 | 201/26565 | 1676/393138 | 1.52(1.31-1.76) | <.001 |
| **Hypothalamic-pituitary-gonadal axis** | No | 1310/420168 | 124/26633 | 1186/393535 | 1.39(1.15-1.67) | <.001 |
| **Diseases of thyroid gland** | No | 21662/394229 | 1852/23982 | 19810/370247 | 1.22(1.16-1.28) | <.001 |
| **Diseases of pancreatic gland** | No | 26309/388689 | 2235/23471 | 24074/365218 | 1.17(1.12-1.23) | <.001 |
| **Diseases of parathyroid gland** | No | 1561/420135 | 146/26617 | 1415/393518 | 1.25(1.06-1.49) | 0.01 |
| **Diseases of hypothalamic-pituitary gland** | No | 938/420773 | 96/26686 | 842/394087 | 1.45(1.17-1.79) | <.001 |
| **Diseases of adrenal gland** | No | 1029/420748 | 111/26673 | 918/394075 | 1.54(1.26-1.87) | <.001 |
| **Diseases of genital gland** | No | 427/421251 | 36/26740 | 391/394511 | 1.38(0.98-1.95) | 0.067 |
| **Hypothyroidism** | No | 18707/398812 | 1627/24394 | 17080/374418 | 1.23(1.17-1.29) | <.001 |
| **Hyperthyroidism** | No | 2529/418604 | 268/26415 | 2261/392189 | 1.46(1.28-1.66) | <.001 |
| **Thyroiditis** | No | 335/421439 | 32/26746 | 303/394693 | 1.47(1.02-2.13) | 0.038 |
| **Type 2 diabetes** | No | 25061/391223 | 2161/23732 | 22900/367491 | 1.18(1.13-1.24) | <.001 |
| **Hypoparathyroidism** | No | 90/421812 | 9/26792 | 81/395020 | 1.32(0.66-2.63) | 0.438 |
| **Hyperparathyroidism** | No | 1507/420203 | 142/26621 | 1365/393582 | 1.26(1.06-1.50) | 0.008 |
| **Hyperpituitarism** | No | 598/421214 | 59/26734 | 539/394480 | 1.34(1.03-1.76) | 0.032 |
| **Hypopituitarism** | No | 371/421439 | 39/26751 | 332/394688 | 1.57(1.13-2.20) | 0.008 |
| **Cushing syndrome** | No | 80/421813 | 8/26791 | 72/395022 | 1.44(0.69-3.00) | 0.335 |
| **Hyperaldosteronism** | No | 79/421795 | 10/26786 | 69/395009 | 2.15(1.10-4.19) | 0.025 |
| **Ovarian dysfunction** | No | 206/421536 | 14/26766 | 192/394770 | 1.16(0.67-2.00) | 0.603 |
| **Testicular dysfunction** | No | 221/421633 | 22/26775 | 199/394858 | 1.61(1.03-2.51) | 0.035 |

^$^Cox model was adjusted for sex, age, BMI, Townsend deprivation index, employment, education, ethnicity, physical activity, smoking status, drinking status, adherence to a healthy diet, social isolation, and C-reactive protein.

Abbreviations: HR: Hazard ratio, CI: Confidence interval, BMI: body mass index.

Table S18 HR (95% CI) of any endocrine disease associated with total and individual gastrointestinal diseases according to baseline type 2 diabetes stratified by age, sex, body mass index, smoking status, and drinking status^$^

| **Outcomes** | **Male** |  | **Female** |  |  | **Age 60≤ years** | | **Age >60 years** | |  |
| --- | --- | --- | --- | --- | --- | --- | --- | --- | --- | --- |
|  | **HR (95% CI)** | **P value** | **HR (95% CI)** | **P value** | **P-interaction** | **HR (95% CI)** | **P value** | **HR (95% CI)** | **P value** | **P-interaction** |
| **Any gastrointestinal diseases** | 1.48(1.42-1.54) | <.001 | 1.48(1.42-1.53) | <.001 | <.001 | 1.60(1.53-1.67) | <.001 | 1.41(1.36-1.46) | <.001 | <.001 |
| **Non-neoplastic diseases** | 1.53(1.47-1.60) | <.001 | 1.49(1.43-1.54) | <.001 | <.001 | 1.62(1.55-1.69) | <.001 | 1.44(1.39-1.49) | <.001 | <.001 |
| **Gastric ulcer** | 1.53(1.34-1.74) | <.001 | 1.57(1.39-1.77) | <.001 | 0.061 | 1.54(1.33-1.77) | <.001 | 1.57(1.40-1.76) | <.001 | <.001 |
| **Duodenal ulcer** | 1.37(1.17-1.60) | <.001 | 1.63(1.35-1.97) | <.001 | <.001 | 1.58(1.29-1.93) | <.001 | 1.40(1.20-1.63) | <.001 | <.001 |
| **Gastritis and duodenitis** | 1.55(1.46-1.65) | <.001 | 1.59(1.52-1.67) | <.001 | <.001 | 1.63(1.54-1.73) | <.001 | 1.55(1.47-1.63) | <.001 | <.001 |
| **Dyspepsia** | 1.33(1.15-1.53) | <.001 | 1.19(1.07-1.32) | 0.001 | <.001 | 1.30(1.14-1.47) | <.001 | 1.19(1.07-1.34) | 0.002 | <.001 |
| **Crohn disease** | 1.19(0.86-1.66) | 0.296 | 1.35(1.05-1.73) | 0.02 | 0.014 | 1.38(1.04-1.83) | 0.023 | 1.21(0.91-1.61) | 0.191 | 0.309 |
| **Ulcerative colitis** | 1.25(1.00-1.57) | 0.055 | 1.27(1.03-1.57) | 0.023 | 0.016 | 1.30(1.03-1.63) | 0.027 | 1.23(1.00-1.51) | 0.048 | 0.61 |
| **Irritable bowel syndrome** | 1.06(0.89-1.27) | 0.512 | 1.49(1.37-1.62) | <.001 | <.001 | 1.51(1.36-1.68) | <.001 | 1.29(1.15-1.44) | <.001 | 0.41 |
| **Intestinal malabsorption** | 1.99(1.55-2.55) | <.001 | 1.69(1.41-2.02) | <.001 | <.001 | 2.05(1.69-2.50) | <.001 | 1.57(1.27-1.95) | <.001 | <.001 |
| **Gastro-intestinal haemorrhage** | 1.43(1.32-1.54) | <.001 | 1.41(1.31-1.53) | <.001 | 0.008 | 1.45(1.33-1.58) | <.001 | 1.39(1.30-1.49) | <.001 | <.001 |
| **Neoplastic diseases** | 0.91(0.81-1.03) | 0.156 | 1.04(0.91-1.19) | 0.579 | <.001 | 1.00(0.85-1.18) | 0.988 | 0.96(0.86-1.07) | 0.494 | <.001 |
| **Gastric cancer** | 1.18(0.92-1.51) | 0.187 | 1.02(0.70-1.50) | 0.91 | <.001 | 0.83(0.53-1.30) | 0.416 | 1.27(1.00-1.61) | 0.05 | <.001 |
| **Small intestine cancer** | 0.79(0.44-1.42) | 0.43 | 1.45(0.89-2.38) | 0.139 | 0.052 | 0.87(0.42-1.79) | 0.699 | 1.21(0.77-1.89) | 0.405 | <.001 |
| **Colorectal cancer** | 0.86(0.74-0.99) | 0.041 | 1.00(0.86-1.16) | 0.991 | <.001 | 1.03(0.86-1.23) | 0.767 | 0.89(0.78-1.01) | 0.063 | <.001 |

| **Outcomes** | **Ever smoker** | | **Non smoker** | |  | **Current drinker** | | **Non-current drinker** | |  |
| --- | --- | --- | --- | --- | --- | --- | --- | --- | --- | --- |
|  | **HR (95% CI)** | **P value** | **HR (95% CI)** | **P value** | **P-interaction** | **HR (95% CI)** | **P value** | **HR (95% CI)** | **P value** | **P-interaction** |
| **Any gastrointestinal diseases** | 1.45(1.40-1.51) | <.001 | 1.52(1.46-1.58) | <.001 | <.001 | 1.48(1.44-1.53) | <.001 | 1.44(1.31-1.58) | <.001 | <.001 |
| **Non-neoplastic diseases** | 1.48(1.42-1.54) | <.001 | 1.55(1.48-1.61) | <.001 | <.001 | 1.51(1.47-1.56) | <.001 | 1.46(1.33-1.61) | <.001 | <.001 |
| **Gastric ulcer** | 1.43(1.28-1.61) | <.001 | 1.72(1.51-1.97) | <.001 | <.001 | 1.52(1.39-1.67) | <.001 | 1.73(1.33-2.25) | <.001 | 0.633 |
| **Duodenal ulcer** | 1.41(1.21-1.65) | <.001 | 1.59(1.31-1.93) | <.001 | <.001 | 1.42(1.25-1.62) | <.001 | 1.86(1.26-2.73) | 0.002 | 0.869 |
| **Gastritis and duodenitis** | 1.51(1.43-1.59) | <.001 | 1.66(1.57-1.76) | <.001 | <.001 | 1.58(1.52-1.65) | <.001 | 1.48(1.30-1.69) | <.001 | 0.075 |
| **Dyspepsia** | 1.18(1.05-1.33) | 0.005 | 1.29(1.14-1.45) | <.001 | <.001 | 1.24(1.14-1.36) | <.001 | 1.15(0.86-1.53) | 0.345 | 0.302 |
| **Crohn disease** | 1.02(0.77-1.35) | 0.905 | 1.73(1.30-2.30) | <.001 | <.001 | 1.22(0.98-1.51) | 0.074 | 1.87(1.07-3.27) | 0.029 | 0.883 |
| **Ulcerative colitis** | 1.13(0.92-1.38) | 0.249 | 1.47(1.17-1.86) | 0.001 | <.001 | 1.32(1.12-1.54) | <.001 | 0.72(0.39-1.35) | 0.305 | 0.531 |
| **Irritable bowel syndrome** | 1.32(1.19-1.48) | <.001 | 1.46(1.31-1.62) | <.001 | <.001 | 1.36(1.26-1.48) | <.001 | 1.59(1.24-2.03) | <.001 | <.001 |
| **Intestinal malabsorption** | 1.46(1.17-1.83) | <.001 | 2.09(1.72-2.53) | <.001 | 0.274 | 1.79(1.54-2.08) | <.001 | 1.77(1.06-2.99) | 0.031 | 0.331 |
| **Gastro-intestinal haemorrhage** | 1.36(1.26-1.46) | <.001 | 1.51(1.39-1.64) | <.001 | <.001 | 1.41(1.33-1.49) | <.001 | 1.55(1.30-1.85) | <.001 | 0.002 |
| **Neoplastic diseases** | 0.94(0.83-1.06) | 0.291 | 1.02(0.89-1.18) | 0.741 | <.001 | 0.97(0.88-1.07) | 0.54 | 1.03(0.72-1.47) | 0.886 | 0.014 |
| **Gastric cancer** | 1.11(0.85-1.43) | 0.447 | 1.18(0.84-1.67) | 0.34 | <.001 | 1.10(0.88-1.37) | 0.401 | 1.52(0.78-2.98) | 0.22 | 0.528 |
| **Small intestine cancer** | 1.07(0.66-1.75) | 0.782 | 1.09(0.60-1.97) | 0.783 | 0.733 | 1.16(0.80-1.70) | 0.434 | NA | 0.998 | 0.88 |
| **Colorectal cancer** | 0.89(0.77-1.02) | 0.085 | 0.99(0.84-1.16) | 0.891 | <.001 | 0.93(0.84-1.04) | 0.198 | 0.90(0.59-1.37) | 0.615 | 0.02 |

| **Outcomes** | **Obesity** |  | **Non-obesity** | |  |
| --- | --- | --- | --- | --- | --- |
|  | **HR (95% CI)** | **P value** | **HR (95% CI)** | **P value** | **P-interaction** |
| **Any gastrointestinal diseases** | 1.49(1.43-1.54) | <.001 | 1.47(1.41-1.54) | <.001 | 0.001 |
| **Non-neoplastic diseases** | 1.51(1.46-1.56) | <.001 | 1.50(1.43-1.57) | <.001 | 0.004 |
| **Gastric ulcer** | 1.54(1.38-1.71) | <.001 | 1.62(1.40-1.88) | <.001 | <.001 |
| **Duodenal ulcer** | 1.37(1.17-1.60) | <.001 | 1.67(1.37-2.03) | <.001 | 0.929 |
| **Gastritis and duodenitis** | 1.58(1.50-1.65) | <.001 | 1.60(1.50-1.70) | <.001 | 0.019 |
| **Dyspepsia** | 1.29(1.15-1.44) | <.001 | 1.18(1.04-1.35) | 0.012 | <.001 |
| **Crohn disease** | 1.32(1.02-1.71) | 0.036 | 1.24(0.90-1.71) | 0.182 | 0.002 |
| **Ulcerative colitis** | 1.15(0.94-1.42) | 0.18 | 1.44(1.15-1.81) | 0.002 | 0.013 |
| **Irritable bowel syndrome** | 1.41(1.28-1.56) | <.001 | 1.35(1.19-1.53) | <.001 | 0.991 |
| **Intestinal malabsorption** | 1.73(1.40-2.14) | <.001 | 1.80(1.47-2.20) | <.001 | <.001 |
| **Gastro-intestinal haemorrhage** | 1.37(1.28-1.47) | <.001 | 1.51(1.38-1.66) | <.001 | <.001 |
| **Neoplastic diseases** | 0.98(0.87-1.09) | 0.694 | 0.96(0.82-1.11) | 0.566 | <.001 |
| **Gastric cancer** | 1.06(0.82-1.37) | 0.629 | 1.29(0.90-1.84) | 0.166 | <.001 |
| **Small intestine cancer** | 0.94(0.57-1.54) | 0.799 | 1.36(0.76-2.45) | 0.3 | 0.075 |
| **Colorectal cancer** | 0.95(0.84-1.08) | 0.44 | 0.89(0.75-1.05) | 0.171 | 0.026 |

^$^Cox model was adjusted for sex, age, BMI, Townsend deprivation index, employment, education, ethnicity, physical activity, smoking status, drinking status, adherence to a healthy diet, social isolation, and C-reactive protein except for stratified factors.

Abbreviations: HR: Hazard ratio, CI: Confidence interval, BMI: body mass index.

Table S19 HR (95% CI) of any endocrine disease associated with total and individual gastrointestinal diseases according to medication use^$^

| **Outcomes** | **medication use** | **Case No./control No.** | | | **HR (95% CI)** | **P value** |
| --- | --- | --- | --- | --- | --- | --- |
|  |  | **Total** | **With**  **endocrine disease** | **Without**  **endocrine disease** |  |  |
| **Any gastrointestinal diseases** | Yes | 12903/29815 | 4566/7708 | 8337/22107 | 1.33(1.28-1.38) | <.001 |
| **Non-neoplastic diseases** | Yes | 12580/30375 | 4496/7910 | 8084/22465 | 1.34(1.29-1.39) | <.001 |
| **Gastric ulcer** | Yes | 1099/48996 | 489/15588 | 610/33408 | 1.45(1.29-1.64) | <.001 |
| **Duodenal ulcer** | Yes | 552/49695 | 248/15917 | 304/33778 | 1.38(1.16-1.64) | <.001 |
| **Gastritis and duodenitis** | Yes | 6196/41446 | 2503/12297 | 3693/29149 | 1.40(1.33-1.47) | <.001 |
| **Dyspepsia** | Yes | 1286/48167 | 472/15359 | 814/32808 | 1.23(1.09-1.38) | <.001 |
| **Crohn disease** | Yes | 249/50104 | 81/16182 | 168/33922 | 0.95(0.72-1.24) | 0.695 |
| **Ulcerative colitis** | Yes | 430/49742 | 147/16040 | 283/33702 | 1.05(0.86-1.29) | 0.639 |
| **Irritable bowel syndrome** | Yes | 1524/48519 | 612/15431 | 912/33088 | 1.43(1.29-1.59) | <.001 |
| **Intestinal malabsorption** | Yes | 492/49842 | 173/16047 | 319/33795 | 1.26(1.04-1.52) | 0.017 |
| **Gastro-intestinal haemorrhage** | Yes | 2747/47261 | 1167/14855 | 1580/32406 | 1.37(1.26-1.48) | <.001 |
| **Neoplastic diseases** | Yes | 1174/49042 | 416/15729 | 758/33313 | 0.99(0.88-1.12) | 0.894 |
| **Gastric cancer** | Yes | 202/50350 | 89/16252 | 113/34098 | 1.27(0.96-1.69) | 0.097 |
| **Small intestine cancer** | Yes | 55/50513 | 25/16328 | 30/34185 | 1.39(0.80-2.39) | 0.242 |
| **Colorectal cancer** | Yes | 940/49323 | 314/15860 | 626/33463 | 0.92(0.80-1.06) | 0.271 |

| **Outcomes** | **medication use** | **Case No./control No.** | | | **HR (95% CI)** | **P value** |
| --- | --- | --- | --- | --- | --- | --- |
|  |  | **Total** | **With**  **endocrine disease** | **Without**  **endocrine disease** |  |  |
| **Any gastrointestinal diseases** | No | 85075/325065 | 1043/2155 | 84032/322910 | 1.40(1.32-1.49) | <.001 |
| **Non-neoplastic diseases** | No | 81503/330313 | 1026/2207 | 80477/328106 | 1.43(1.35-1.52) | <.001 |
| **Gastric ulcer** | No | 5519/444100 | 93/3996 | 5426/440104 | 1.32(1.08-1.63) | 0.008 |
| **Duodenal ulcer** | No | 3144/446720 | 55/4059 | 3089/442661 | 1.37(1.05-1.79) | 0.022 |
| **Gastritis and duodenitis** | No | 34812/401659 | 532/3267 | 34280/398392 | 1.44(1.32-1.56) | <.001 |
| **Dyspepsia** | No | 9085/435800 | 127/3912 | 8958/431888 | 1.33(1.12-1.59) | 0.001 |
| **Crohn disease** | No | 1465/449203 | 27/4113 | 1438/445090 | 1.72(1.17-2.52) | 0.006 |
| **Ulcerative colitis** | No | 2713/447007 | 35/4086 | 2678/442921 | 1.24(0.89-1.73) | 0.209 |
| **Irritable bowel syndrome** | No | 9297/439887 | 123/3976 | 9174/435911 | 1.21(1.01-1.45) | 0.035 |
| **Intestinal malabsorption** | No | 2393/448390 | 31/4116 | 2362/444274 | 1.39(0.97-1.98) | 0.072 |
| **Gastro-intestinal haemorrhage** | No | 17459/431493 | 293/3772 | 17166/427721 | 1.50(1.33-1.68) | <.001 |
| **Neoplastic diseases** | No | 8920/440468 | 94/4012 | 8826/436456 | 0.91(0.74-1.12) | 0.362 |
| **Gastric cancer** | No | 1247/450366 | 12/4148 | 1235/446218 | 0.69(0.39-1.23) | 0.21 |
| **Small intestine cancer** | No | 431/451285 | 5/4154 | 426/447131 | 0.93(0.38-2.24) | 0.866 |
| **Colorectal cancer** | No | 7447/442153 | 80/4037 | 7367/438116 | 0.95(0.76-1.19) | 0.665 |

^$^Cox model was adjusted for sex, age, BMI, Townsend deprivation index, employment, education, ethnicity, physical activity, smoking status, drinking status, adherence to a healthy diet, social isolation, and C-reactive protein.

Abbreviations: HR: Hazard ratio, CI: Confidence interval, BMI: body mass index.
